# Plasma‐Activated Water Primes Black Rice Starch for Amplified Hydrothermal Modification: Structural Insights and Enhanced Digestibility

**DOI:** 10.1111/1750-3841.71534

**Published:** 2026-10-07

**Authors:** Raphael Lucas Jacinto Almeida, Newton Carlos Santos, João Vitor Fonseca Feitoza, Iêda Letícia de Souza Ferreira, Raphael da Silva Eduardo, André Miranda da Silva, Cecilia Elisa Sousa Muniz, Ellen Cristina Nabiça Rodrigues, Alison Bruno Borges de Sousa, Lícia Amazonas Calandrini Braga, Adriano Cesar Calandrini Braga, Victor Herbert de Alcântara Ribeiro, Ynis Thaciane Felix da Silva, Martha Maria Gomes Nóbrega, Ana Carla Oliveira de Brito, Virgínia Mirtes de Alcantara Silva

**Affiliations:** ^1^ Department of Chemical Engineering Federal University of Rio Grande do Norte Natal Rio Grande do Norte Brazil; ^2^ Department of Food Engineering Federal University of Campina Grande Campina Grande Paraíba Brazil; ^3^ Institute of Rural Development Universidade da Integração Internacional da Lusofonia Afro‐Brasileira Redenção Ceará Brazil; ^4^ Department of Chemical Engineering Federal University of Campina Grande Campina Grande Paraíba Brazil; ^5^ Department of Food Engineering Federal Institute of Education, Science and Technology of Piaui Uruçuí Piaui Brazil; ^6^ Department of Agroindustry Federal Institute of Education, Science and Technology of Pernambuco Vitória de Santo Antão Pernambuco Brazil; ^7^ Department of Agroindustry Federal Institute of Education, Science and Technology of Pará Castanhal Pará Brazil; ^8^ Department of Food Technology State University of Pará Belém Pará Brazil; ^9^ Department of Agricultural Sciences Federal University of Paraiba Bananeiras Paraíba Brazil; ^10^ Department of Chemical Engineering Federal University of Paraiba João Pessoa Paraíba Brazil; ^11^ Department of Materials Engineering Federal University of Campina Grande Campina Grande Paraíba Brazil; ^12^ Department of Food Quality and Safety Management State University of Campinas Campinas São Paulo Brazil

**Keywords:** functional foods, physical processing, sustainable modification

## Abstract

This study evaluated the ability of plasma‐activated water (PAW) to modulate black rice starch response to autoclaving. Six formulations were prepared: native starch (Native), starch treated with PAW for 30 or 60 min (P30, P60), starch autoclaved only (AC), and starch subjected to PAW for 30 or 60 min prior to autoclaving (P30AC, P60AC). PAW pretreatment intensified autoclaving‐induced depolymerization, as reflected by pronounced reductions in the hydrodynamic radius (Rh) of amylose and amylopectin chains. This molecular breakdown was accompanied by a redistribution of linear glucan chains toward shorter fractions (DP 6–12 increasing to 20.17%) and a decrease in apparent amylose content (up to 16.76%). Solubility increased nearly fivefold in P60AC (31.57%), while relative crystallinity declined from 25.18% to 19.68%, indicating partial loss of ordered lamellae. Gelatinization enthalpy (ΔH) was markedly reduced to 5.32 J g^−^^1^, and pasting viscosities were strongly suppressed. Morphological analysis revealed progressive granule swelling, with average diameter increasing from 6.89 µm (native) to 12.50 µm (P60AC), reflecting extensive structural disorganization and granule fusion induced by the combined action of PAW and autoclaving. Regarding freeze‐thaw stability, combined treatments exhibited the lowest syneresis values throughout five cycles, indicating superior gel water retention capacity. The results demonstrate that PAW acts as an effective non‐thermal priming step that amplifies the structural and functional impact of autoclaving on black rice starch, offering a sustainable approach for tailoring starch properties with enhanced digestibility and improved gel stability.

## 1 Introduction

Black rice is distinguished from other cereals not only by its dark pigmentation but also by the concentration of anthocyanins and other phenolic compounds in its outer grain layers. These bioactive compounds contribute to strong antioxidant activity and have been associated with protective effects against oxidative stress‐related disorders (Bhat et al. 2020). From a nutritional and technological standpoint, however, the dominant component of black rice remains starch, which accounts for nearly 90% of its dry matter and governs both energy delivery and the textural and sensory properties of cooked grains (Lin et al. 2024).

These structural constraints manifest as limited swelling and solubility in cold water, low enzymatic susceptibility, and restricted functional versatility in their native granular state (An et al. 2025). Upon gelatinization, these same starches exhibit poor shear stability and a strong tendency toward retrogradation, which further hinder their performance in processed food systems (Wang et al. 2025). Consequently, physical modification approaches have attracted increasing attention as environmentally compatible routes to tailor starch functionality without introducing chemical residues or generating secondary pollution streams (Almeida et al. 2024). Within this framework, sequential or combined treatments have been proposed as a way to enhance the extent of molecular and granular disorganization beyond what is achievable by single treatments alone, while avoiding the use of chemical reagents and reducing energy consumption compared to more intensive processing methods (Bai et al. 2021).

Among emerging non‐thermal technologies, plasma‐activated water (PAW) offers a unique means of introducing chemical reactivity into aqueous systems without direct exposure of the material to plasma. The interaction between atmospheric plasma and water produces a metastable liquid phase containing a complex mixture of oxidizing species capable of interacting with biopolymer chains and interfaces (Thirumdas et al. 2018). Rather than simply modifying starch through bulk heating, PAW alters the chemical environment surrounding starch granules, enabling localized oxidation and rearrangement of macromolecular structures (Sifuentes‐Nieves et al. 2024). Recent studies have shown that when PAW is combined with hydrothermal treatments, the resulting starch exhibits altered hydration behavior, reduced retrogradation, and modified digestibility, as demonstrated for quinoa starch and wheat flour systems (Yan et al. 2022; Liu et al. 2026).

Autoclaving represents a distinct class of hydrothermal processing in which starch is subjected to elevated temperature and pressure in the presence of excess water, promoting complete gelatinization followed by molecular reorganization during cooling. This process favors the reassociation of amylose chains into more stable crystalline arrangements, which are commonly associated with the formation of type RS3 resistant starch and changes in rheological behavior (Ashwar et al. 2016; Kumari and Sit 2023; Junden et al. 2025). While PAW has been shown to enhance the effects of milder heat‐moisture treatments, such as annealing, its interaction with pressurized hydrothermal processing remains largely unexplored, despite early evidence of synergistic effects in waxy corn starch systems (Yan et al. 2020).

Based on these considerations, it is proposed that plasma‐activated water may serve not merely as a reaction medium but as a chemical modulator capable of altering the surface chemistry and hydration behavior of black rice starch prior to hydrothermal processing. Such preconditioning is expected to influence chain mobility, depolymerization pathways, and the subsequent reassembly of glucan chains under autoclave conditions, leading to structural outcomes that differ from those obtained by thermal treatment alone. These structural modifications are particularly relevant from a nutritional perspective, as the organization of starch at the molecular and supramolecular levels governs its digestibility and glycemic response. By reshaping chain length distribution and crystalline packing, combined PAW and autoclaving treatments have the potential to shift the balance between rapidly digestible, slowly digestible, and resistant starch fractions, thereby enabling the development of black rice–based ingredients with tailored metabolic impacts. In this context, the present study examines the combined application of plasma‐activated water and autoclaving as a dual modification strategy for black rice starch. By integrating a non‐thermal chemical activation step with high‐pressure hydrothermal processing, this work aims to elucidate how molecular, structural, and functional attributes of black rice starch can be tailored to support its use in advanced and health‐oriented food formulations.

## 2 Materials and Methods

### 2.1 Obtaining Biological Material and Chemical Reagents

Black rice grains (Urbano) were sourced from local retail markets in Natal (RN, Brazil). The enzymes isoamylase (280 U/mg protein, from *Pseudomonas* sp.) and amyloglucosidase (3260 U/mL, from *Aspergillus niger*) were supplied by Megazyme (Bray, Ireland). Porcine pancreatic α‐amylase (Type VI‐B, with an activity of 290 U/mg solid) was purchased from Merck (Darmstadt, Germany). All other reagents, including dimethylsulfoxide (DMSO), 3,5‐dinitrosalicylic acid (DNS), sodium metabisulfite (97%), iodine (resublimed, 99.8%) and potassium iodide (76%), were acquired from Synth (Diadema, Brazil).

### 2.2 Starch Isolation Procedure

Starch extraction followed a previously described wet‐milling approach (Almeida et al., 2019), with adaptations for black rice. Grains were first steeped in a 0.5% (w/w) sodium metabisulfite solution (1:2 grain‐to‐solution ratio) for 48 h at room temperature to soften the endosperm and inactivate oxidative enzymes. After rinsing under running tap water for 3 min, the softened grains were blended with distilled water (1:2 w/w) in an industrial blender (Kohlbach, KM42A, Brazil) at 10,000 rpm for 5 min. The resulting slurry was filtered through a 0.1‐mm organza mesh, and the retained residue was re‐homogenized once more to maximize starch recovery. The combined filtrate was allowed to settle for 24 h at 7°C, after which the supernatant was discarded. The precipitated starch layer was finally dried in a convection oven (7Lab, Bio SEDi‐C 85L, Brazil) at 55°C for 400 min. After drying, the starch samples were ground in a disc mill (BOTINI, B55, Brazil) to obtain a fine, homogeneous powder, following the procedure standardized in previous studies (Almeida et al. 2021). The ground starch was stored in airtight containers at room temperature (25°C), protected from light and moisture.

### 2.3 Preparation of Plasma‐Activated Water (PAW)

PAW was produced using an atmospheric pressure plasma jet system (operating at 750 W and approximately 25 kHz). In each batch, 100 mL of distilled water was placed in a cylindrical glass vessel (12 cm height × 2.7 cm diameter). The plasma nozzle was positioned 25 mm above the water surface, and the discharge was sustained for 2 min (120 s). This protocol has been validated in previous starch modification studies (Yan et al. 2020). The resulting PAW was used immediately after generation to minimize decay of reactive species.

### 2.4 Single and Sequential Treatments

Six different starch formulations were prepared to evaluate the individual and combined effects of plasma‐activated water (PAW) and autoclaving on black rice starch properties. Native starch, used as the control reference, remained untreated. For the PAW‐only treatments, native starch was mixed with PAW at a starch‐to‐PAW ratio of 1:8 (w/v) and allowed to interact under continuous stirring for either 30 min (P30) or 60 min (P60) at room temperature (25°C), to assess the effect of PAW exposure alone and the influence of prolonged exposure time. For the autoclaving‐only treatment (AC), native starch (25.0 g) was suspended in 200 mL distilled water and autoclaved (GENERALMED, CS50, Brazil) at 121°C and 1.1 bar for 3 min, following a previously established protocol (Almeida et al. 2021). Autoclaving was selected as the thermal treatment because it represents a widely used hydrothermal processing condition in the food industry, simulating pressurized cooking conditions that promote molecular reorganization and gelatinization depending on the operational parameters (Ashwar et al. 2016). Furthermore, autoclaving provides a controlled and reproducible high‐temperature, high‐pressure environment that enables the evaluation of PAW pretreatment effects on starch susceptibility to thermal degradation, as the pressurized conditions facilitate water penetration and enhance molecular mobility within the granule matrix (Faridah et al. 2022).

For the combined treatments, starch was first exposed to PAW for either 30 min (P30AC) or 60 min (P60AC), and then the entire suspension was autoclaved under the same conditions as the AC treatment (121°C and 1.1 bar for 3 min), to evaluate the combined effect of PAW pretreatment followed by autoclaving.

After each treatment, the starch suspensions were centrifuged (PROLAB, CDM‐16000‐110 V, Brazil) at 4000 rpm for 10 min to remove excess water and facilitate drying. The supernatant was carefully discarded, and the precipitated starch fraction was collected. The centrifugation step was applied to all autoclaved samples (AC, P30AC, and P60AC) to separate the gelatinized starch from the excess aqueous medium, as well as to the PAW‐treated samples (P30 and P60) to recover the starch after the hydration period. The recovered starch pellets were then dried (7Lab, Bio SEDi C 85L, Brazil) at 55°C until a constant mass was reached. At the end of the processes, the yield was calculated based on the ratio of the starch mass before and after the treatments.

### 2.5 Carbonyl and Carboxyl Content

Carbonyl content was determined following the hydroxylamine method described by Zhang et al. (2023) with minor modifications. Starch samples (0.5 g) were dispersed in 25 mL of distilled water and heated at 80°C for 30 min under stirring. The suspension was then cooled to 40°C, and the pH was adjusted to 3.2 by dropwise addition of 0.1 M HCl. Subsequently, 1.88 mL of 0.25 M hydroxylamine hydrochloride solution was added, and the mixture was incubated for 4 h at 40°C. At the end of the incubation period, the pH was readjusted to 3.2, and the carbonyl content was calculated using Equation (1), expressed as millimoles of carbonyl groups per 100 g of glucose equivalents.

(1) $$\begin{array}{ccc} & & \mathrm{Carbonyl}\;\left(C\;=\;O/100\;g\;\mathrm{glucose}\right)=\\ & & \frac{\left[\left(\mathrm{Vb}1-\mathrm{Vs}1\right)\times C\left(\mathrm{HCl}\right)\times 0.028\times 100\right]}{m}\; \end{array}$$

where: Vb1 is the volume of HCl consumed in the blank (mL); Vs1 is the volume of HCl for starch (mL); C(HCl) is the molar concentration of HCl (0.05 mol/L); and 0.028 is the molecular weight of carbonyl/1000 (g/mmol).

Carboxyl content was determined according to the protocol of Lima et al. (2020). Starch samples (0.5 g, dry basis) were dispersed in 4.2 mL of 0.1 M HCl and centrifuged (PROLAB, CDM‐16000‐110 V, Brazil) at 4000 rpm for 5 min. The supernatant was discarded, and the pellet was washed with 50 mL of distilled water. The suspension was then heated in a water bath at 80°C for 20 min, followed by pH adjustment to 8.3 using 0.01 M NaOH under continuous monitoring. Carboxyl content was calculated using Equation (2), expressed as millimoles of carboxyl groups per 100 g of glucose equivalents.

(2) $$\begin{array}{ccc} & & \mathrm{Carboxyl}\;\left(\mathrm{COOH}/100\;g\;\mathrm{glucose}\right)=\\ & & \frac{\left[\left(\mathrm{Vs}2-\mathrm{Vb}2\right)\times C\left(\mathrm{NaOH}\right)\times 0.045\times 100\right]}{m}\; \end{array}$$

where: Vs2 is the volume of NaOH required for starch (mL); Vb2 is the volume of NaOH consumed in the blank (mL); C(NaOH) is the molar concentration of NaOH (0.01 mol/L); 0.045 is the molecular weight of carboxyl/1000 (g/mmol); and *m* is the mass of starch on a dry basis (g).

### 2.6 Hydrodynamic Radius Measurement

The hydrodynamic radius (Rh) of starch samples was measured using dynamic light scattering (DLS) on a Zetasizer Nano ZS instrument (Malvern Panalytical, Worcestershire, UK). The system was equipped with a 4 mW helium‐neon laser operating at 632.8 nm, and scattering was detected at an angle of 173° (backscatter configuration).

Starch dispersions were prepared at a concentration of 1 mg/mL in deionized water. The suspensions were stirred magnetically for 2 h at 25°C to ensure proper hydration while avoiding gelatinization. Prior to analysis, each dispersion was passed through a 0.45 µm PVDF syringe filter (Millipore, Burlington, MA, USA) to eliminate dust and large aggregates that could interfere with the light scattering signal. The filtered samples were then transferred to disposable polystyrene cuvettes for measurement.

All DLS measurements were conducted at a controlled temperature of 25.0 ± 0.1°C. Each sample was analyzed in triplicate using three independently prepared dispersions, and for each replicate, 12 consecutive runs of 10 s were recorded. The refractive index of starch was set to 1.53, while that of the dispersant (water) was 1.33, with a viscosity of 0.8872 cP at 25°C.

The intensity autocorrelation functions were processed using the cumulants method to extract the z‐average translational diffusion coefficient (D). The hydrodynamic radius (Rh) was subsequently derived from the Stokes‐Einstein relationship (Equation 3):

(3) $$\mathrm{Rh}\;\left(\mathrm{nm}\right)=\frac{\mathrm{kT}}{6\pi \eta D}\;$$

where k is the Boltzmann constant (1.38065 × 10^−^^2^ ^3^ J/K), T is the absolute temperature (298.15 K), *η* is the solvent viscosity (0.8872 × 10^−^^3^ Pa·s for water at 25°C), and *D* is the translational diffusion coefficient (m^2^/s).

### 2.7 Amylose Content

Apparent amylose content was measured by the iodine‐binding method (Nadiha et al., 2010) with minor modifications. Twenty milligrams of starch (dry basis) were dissolved in 8 mL of 90% DMSO and heated at 85°C for 15 min to ensure complete dissolution. The solution was then diluted to 25 mL with distilled water, and a 1 mL aliquot was transferred to a 50 mL volumetric flask. Five milliliters of iodine–potassium iodide solution (2% w/v; final concentrations 2.5 mM I_2_ and 6.5 mM KI) were added, and the volume was made up with distilled water. After 10 min of incubation in the dark to allow color development, the absorbance was recorded at 620 nm using a SP‐2000 UV spectrophotometer (Spectrum, Shanghai, China).

A calibration curve was prepared using pure amylose (Sigma‐Aldrich, St. Louis, MO, USA) at concentrations ranging from 0.01 to 1.0 g/mL. The amylose standards were subjected to the same dissolution and heating conditions as the starch samples (i.e., dissolved in 90% DMSO and heated at 85°C for 15 min) to ensure consistency and accurate quantification of apparent amylose content in the starch samples. The absorbance of each standard was measured at 620 nm following the same iodine‐binding protocol described above. The calibration curve was constructed by plotting absorbance versus amylose concentration, and the apparent amylose content of each starch sample was calculated from the linear regression equation of the standard curve.

### 2.8 Distribution of Linear Glucan Chains After Enzymatic Debranching

The distribution of linear glucan chains after enzymatic debranching was determined by high‐performance anion‐exchange chromatography with pulsed amperometric detection (HPAEC‐PAD) (Jane et al., 1999; Syahariza et al., 2013). Fifty milligrams of starch were dissolved in 1 mL DMSO, followed by precipitation with methanol. The precipitate was re‐dissolved in 9 mL deionized water and heated at 96°C for 1 h with continuous stirring. After cooling to 25°C, 1 mL of 0.1 M acetate buffer (pH 3.5) and isoamylase (from *Pseudomonas* sp.) were added, and the mixture was incubated at 40°C for 48 h in a thermostatic shaking bath to ensure complete debranching of amylopectin.

An ICS‐5000 chromatograph (Thermo Fisher Scientific, USA) equipped with a CarboPac PA200 column (3.0 × 250 mm) was used for separation. The eluents were: (A) 100 mM NaOH and (B) 300 mM NaNO_3_ in 100 mM NaOH. The gradient for eluent B was set as follows: 0 min, 1%; 39 min, 5%; 50 min, 8%; 170 min, 30%; 220 min, 45%, at a constant flow rate of 0.4 mL/min. Chain lengths were expressed as degree of polymerization (DP) and grouped into four fractions: DP 6–12, DP 13–24, DP 25–36, and DP ≥ 37. Since the samples were debranched prior to chromatography, the profiles obtained represent the distribution of linear glucan chains derived from both amylose and amylopectin, rather than the native branch chain length distribution of amylopectin alone.

### 2.9 In Vitro Starch Digestibility and Nutritional Fractions

Enzymatic digestibility was evaluated according to the method described by Guo et al. (2018) with minor modifications. Starch samples (100 mg, dry basis) were placed in glass tubes containing 4 mL of 0.1 M sodium acetate buffer (pH 5.2). The enzymatic reaction was initiated by adding 10 mL of a mixture of α‐amylase (290 U/mL, from porcine pancreas) and amyloglucosidase (100 U/mL, from *Aspergillus niger*), resulting in an enzyme‐to‐starch ratio of approximately 29 U of α‐amylase and 10 U of amyloglucosidase per mg of starch. The tubes were incubated at 37°C under constant magnetic stirring (MYLABOR MS4‐H380‐220 V), (260 rpm) for 120 min.

At selected time points (0, 20, and 120 min), aliquots of 0.5 mL were withdrawn and immediately mixed with 4.5 mL of anhydrous ethanol to terminate the enzymatic reaction. The mixtures were then centrifuged at 4000 rpm for 10 min, and the glucose content in the supernatant was determined using the 3,5‐dinitrosalicylic acid (DNS) method (Miller 1959). A glucose standard curve was prepared using D‐glucose solutions at concentrations ranging from 0 to 1.0 mg/mL. The absorbance was measured at 540 nm using a SP‐2000 UV spectrophotometer (Spectrum, Shanghai, China). Free glucose content (FG) was determined in samples without enzyme addition, representing the initial glucose present in the starch samples. Total starch (TS) was determined as the total glucose released after complete enzymatic hydrolysis of the starch samples using the same enzyme mixture.

Based on the hydrolysis kinetics, rapidly digestible starch (RDS, hydrolyzed within 20 min) (Equation 4), slowly digestible starch (SDS, hydrolyzed between 20 and 120 min) (Equation 5), and resistant starch (RS, remaining after 120 min) (Equation 6) were quantified according to the following equations (Englyst et al. 1992):

(4) $$RDS\;\;\left(\%\right)=\frac{\left(G20-FG\right)\times 0.9}{TS}\;\times 100\%$$

(5) $$SDS\;\;\left(\%\right)=\frac{\left(G120-G20\right)\times 0.9}{TS}\;\times 100\%$$

(6) $$RS\;\;\left(\%\right)=\left(1-RDS-SDS\right)\times 100\%$$

where G20 and G120 are the glucose content (mg) released after 20 and 120 min of hydrolysis, respectively; FG is the free glucose content (mg) before enzymatic treatment; TS is the total starch content (mg); and 0.9 is the conversion factor from glucose to starch. Resistant starch (RS) was defined as the starch fraction that remained undigested after 120 min of enzymatic hydrolysis, as it is not hydrolyzed by α‐amylase and amyloglucosidase under the conditions used.

Total starch (TS) content of each sample was determined following the same enzymatic hydrolysis protocol described above, with modifications for complete starch digestion. Briefly, starch samples (100 mg, dry basis) were dispersed in 4 mL of 0.1 M sodium acetate buffer (pH 5.2) in glass tubes. The enzymatic reaction was initiated by adding 10 mL of the same enzyme mixture (α‐amylase, 290 U/mL; amyloglucosidase, 100 U/mL). The tubes were incubated at 37°C for 24 h under constant magnetic stirring (260 rpm) to ensure complete hydrolysis of all starch fractions, including resistant starch. After 24 h, aliquots of 0.5 mL were withdrawn and immediately mixed with 4.5 mL of anhydrous ethanol to stop the reaction. The mixtures were centrifuged at 4000 rpm for 10 min, and the glucose content in the supernatant was determined using the DNS method (Miller 1959).

### 2.10 Water Solubility Index

Solubility was determined gravimetrically at 25°C according to the method described by Leach et al. (1959) with modifications. Starch samples (2.0 g, dry basis) were dispersed in 30 mL of distilled water in 50 mL centrifuge tubes. The suspensions were continuously stirred using a magnetic stirrer (400 rpm) throughout the 30 min incubation period at 25°C to ensure proper hydration and to prevent sedimentation of starch granules, which could compromise the reliability of the solubility results. After incubation, the suspension was vortexed for 2 min (Netlab, K4528XX, Brazil) to ensure complete dispersion, and then centrifuged at 3000 rpm for 10 min (SOLAB, SL706, Brazil). The supernatant was carefully decanted and transferred to pre‐weighed aluminum crucibles, then evaporated to dryness in an oven at 105°C until constant weight was achieved. Solubility (%) was calculated as the ratio of the mass of dried supernatant to the initial dry starch mass, multiplied by 100, according to the following Equation (7):

(7) $$Solubility\;\;\left(\%\right)=\frac{Msupernatant}{Mstarch}\;\times 100\%$$

where *Msupernatant* is the mass of dried supernatant (g) and *Mstarch* is the initial dry mass of starch (g).

### 2.11 Granule Morphology by Scanning Electron Microscopy (SEM)

Starch samples were mounted on aluminum stubs using double‐sided carbon tape and coated with a thin gold layer (sputter coater). Imaging was performed on a Nano SEM‐450 (FEI Company, USA) at an accelerating voltage of 15 kV and a magnification of 1000×. Average granule diameters were measured from at least 100 granules per sample using ImageJ software.

### 2.12 X‐Ray Diffraction (XRD) and Crystallinity

XRD patterns were recorded on a Rigaku D/max2200pc diffractometer (Japan) with Cu‐Kα radiation (λ = 0.15406 nm), operating at 40 kV and 100 mA. The scanning range was 2θ = 10° to 40°, with a step size of 0.02° and a scan speed of 1°/min. Relative crystallinity was calculated as the ratio of the area of crystalline peaks to the total area of the diffractogram (from 10° to 40°), using the MDI Jade 6.0 software.

### 2.13 Thermal Properties by Differential Scanning Calorimetry (DSC)

Gelatinization parameters were measured using a modulated DSC (2920, TA Instruments, New Castle, DE, USA) according to Zhu et al. (2016). Approximately 12 mg of starch (dry basis) were weighed into aluminum pans, and 28 mg of distilled water were added. The pans were hermetically sealed and equilibrated overnight at room temperature. Samples were heated from 20°C to 200°C at a rate of 10°C/min. An empty pan served as the reference. The onset (*T*_o_), peak (*T*_p_), conclusion (*T*_c_) temperatures and the enthalpy of gelatinization (ΔH) were calculated from the thermograms using TA Universal Analysis software.

### 2.14 Syneresis (Freeze‑Thaw Stability)

Starch pastes (10% w/w) were prepared by dispersing starch in distilled water and heating at 80°C for 12 min in a water bath (TE‐184, TECNAL, Brazil) with constant stirring to ensure complete gelatinization. The pastes were then cooled to room temperature (25°C) and transferred to centrifuge tubes. Approximately 15 g of each paste were weighed into 50 mL centrifuge tubes.

For each freeze‐thaw cycle, the pastes were first frozen at ‐18°C for 24 h in a freezer (CONSUL, Brazil). After freezing, the samples were thawed at 25°C for 2 h in a thermostatic water bath (TE‐184, TECNAL, Brazil) to ensure complete thawing. The thawed pastes were then centrifuged at 3000 rpm for 10 min (SOLAB, SL706, Brazil) to separate the released water. The supernatant was carefully decanted and weighed, and the syneresis percentage was calculated (Equation 8). The entire freezing‐thawing‐centrifugation sequence was repeated five times to evaluate the stability of the gels under repeated freeze‐thaw stress.

(8) $$Syneresis\;\;\left(\%\right)=\frac{Mwater}{Mpaste}\;\times 100\%$$

where *Mwater* is the mass of water released after centrifugation (g) and *Mpaste* is the initial mass of the paste before freezing (g).

### 2.15 Pasting Properties

Pasting characteristics were determined using a Rapid Visco Analyzer (RVA 4500, PerkinElmer, Australia). An aqueous suspension of starch (8% w/w, total weight 28 g) was prepared in the RVA canister and subjected to the following heating‐cooling cycle: equilibration at 50°C for 1 min, heating to 95°C at a rate of 12°C/min, holding at 95°C for 2.5 min, cooling to 50°C at a rate of 12°C/min, and finally holding at 50°C for 2 min. The paddle speed was maintained at 160 rpm throughout the analysis. The following pasting parameters were recorded from the viscosity profile: peak viscosity (PV), minimum viscosity (MV, also known as trough), breakdown (BD = PV − MV), final viscosity (FV), and setback (SB = FV − MV).

### 2.16 Statistical Treatment

The experiment was conducted using a completely randomized design in a 3 × 2 factorial arrangement, combining two factors: plasma‐activated water (PAW) exposure time (0, 30, and 60 min) and autoclaving (without or with autoclaving). This arrangement resulted in six treatment combinations: Native, P30, P60, AC, P30AC, and P60AC. Starch was extracted in a single batch, and aliquots of this batch were independently subjected to each treatment condition. All measurements were performed in triplicate (three independent analytical replicates per treatment), and results are expressed as mean ± standard deviation.

Data were analyzed by Welch's ANOVA, which does not assume homogeneity of variances, followed by the Dunnett‐T3 post‐hoc test for pairwise comparisons among all treatment means, with a confidence level of 95% (α = 0.05). Pearson correlation analysis was performed to evaluate the linear associations among physicochemical, morphological, and structural parameters, thermal and viscoamylographic properties, and the in vitro digestibility of black rice starches. Pearson correlation coefficients (*r*) were calculated for all evaluated variables, and their statistical significance was determined at the 5% level (*p* < 0.05). A correlation matrix was used to graphically represent the strength and direction of the associations between the variables, using OriginPro 9 software (OriginLab, Northampton, MA, USA).

## 3 Results and Discussion

### 3.1 Extraction Yield, Carbonyl and Carboxyl Content

The yields obtained for native black rice starch (58.49%) and for samples treated with plasma‐activated water (P30: 58.08%; P60: 58.22%) were higher than those reported by Ribeiro et al. (2023) for native black rice starch (52.06%) and for starch subjected to hydrothermal modification (44.31%). This difference may be attributed to variations in the extraction method, the cultivar used, and the isolation conditions. Conversely, samples subjected to autoclaving (AC, P30AC, and P60AC) showed significantly lower yields (49.08%–49.45%), which is consistent with the value of 44.31% reported by Ribeiro et al. (2023) following hydrothermal treatment. This reduction is expected, as moist heat treatment promotes partial gelatinization and the loss of soluble material during recovery steps, thereby reducing the final yield. Pre‐treatment with plasma‐activated water (P30 and P60) did not significantly alter the yield compared to native starch, indicating that the plasma‐activated water did not cause additional material loss during the process.

As expected, native starch (Native) and starch subjected only to autoclaving (AC) exhibited no detectable carbonyl or carboxyl groups (Table 1), since both treatments were performed with distilled water and no oxidizing agents were introduced. In contrast, samples exposed to plasma‐activated water (P30 and P60) showed progressive increases in carbonyl and carboxyl contents, reaching 0.45 and 0.22, respectively, for P60. This confirms that the reactive species present in PAW are capable of oxidizing hydroxyl groups at the granule surface, introducing carbonyl and carboxyl functionalities (Zuo et al. 2024). The combined treatments P30AC and P60AC exhibited the highest values (carbonyl: 0.89 and 1.12; carboxyl: 0.51 and 0.68, respectively), reflecting the greater surface area available for oxidation after PAW‐induced erosion and the subsequent thermal degradation that exposes new reactive sites (Gupta et al. 2024). The absence of oxidative groups in the AC sample further supports that the chemical modifications observed in the combined treatments are primarily due to the PAW pretreatment, rather than to the autoclaving step itself.

**TABLE 1:** Physicochemical, morphological, and structural parameters of black rice starch after single and combined physical treatments.

Parameters	Native	P30	P60	AC	P30AC	P60AC
**Yield (%)**	58.49 ± 1.49^A^	58.08 ± 0.24^A^	58.22 ± 0.18^A^	49.08 ± 0.75^B^	49.45 ± 0.42^B^	49.27 ± 0.55^B^
**Carbonyl** **(C═O/100g glucose)**	—	0.28 ± 0.02^D^	0.45 ± 0.03^C^	—	0.89 ± 0.05^B^	1.12 ± 0.06^A^
**Carboxyl** **(COOH/100g glucose)**	—	0.15 ± 0.02^D^	0.22 ± 0.02^C^	—	0.51 ± 0.04^B^	0.68 ± 0.05^A^
**DP 6–12**	18.05 ± 0.12^C^	17.04 ± 0.11^D^	16.96 ± 0.21^D^	19.69 ± 0.12^B^	20.09 ± 0.07^A^	20.17 ± 0.07^A^
**DP 13–24**	45.16 ± 0.16^D^	45.37 ± 0.14^D^	45.55 ± 0.22^D^	47.03 ± 0.15^C^	47.72 ± 0.12^B^	48.12 ± 0.09^A^
**DP 25–36**	16.07 ± 0.21^B^	16.78 ± 0.14^A^	16.92 ± 0.17^A^	14.19 ± 0.21^C^	13.71 ± 0.15^D^	13.69 ± 0.11^D^
**DP ≥ 37**	21.14 ± 0.15^A^	20.81 ± 0.11^B^	20.57 ± 0.07^B^	19.09 ± 0.32^C^	18.48 ± 0.16^D^	18.02 ± 0.13^E^
**Solubility (%)**	6.54 ± 0.37^F^	10.09 ± 0.11^E^	13.36 ± 0.31^D^	22.12 ± 0.28^C^	29.57 ± 0.41^B^	31.57 ± 0.86^A^
**Average diameter (µm)**	6.89 ± 0.11^E^	7.02 ± 0.21^E^	8.43 ± 0.15^D^	10.06 ± 0.23^C^	11.27 ± 0.41^B^	12.50 ± 0.26^A^
**Crystallinity (%)**	25.18 ± 0.71^A^	23.40 ± 0.33^B^	23.03 ± 0.49^B^	21.81 ± 0.42^C^	20.57 ± 0.55^D^	19.68 ± 0.27^D^

*Note*: values are expressed (average ± standard deviation). Different superscript capital letters indicate that there is a significant difference (*p* < 0.05) between the formulations. Native, black rice native starch; P30, black rice starch hydrated with plasma‐activated water (PAW) for 30 min; P60, black rice starch hydrated with plasma‐activated water (PAW) for 60 min; AC, native starch suspended in distilled water and autoclaved (121°C, 3 min); P30AC, starch suspended in plasma‐activated water for 30 min and autoclaved for 3 min; P60AC, starch suspended in plasma‐activated water for 60 min and autoclaved for 3 min.

O oxidative mechanism triggered by PAW can be traced across multiple levels of starch structure, providing a coherent explanation for the observed changes. PAW contains reactive oxygen and nitrogen species (reactive oxygen and nitrogen species, RONS)—including hydroxyl radicals, hydrogen peroxide, ozone, nitrates, and nitrites—that decrease pH and increase the oxidation‐reduction potential of the aqueous medium (Sifuentes‐Nieves et al. 2024). These species promote the oxidation of C–C/C–H and C–O bonds at the granule surface into carboxyl groups (Okyere et al. 2022), which is directly evidenced by the progressive increase in carbonyl and carboxyl contents observed in P30 and P60 samples (Table 1). This surface oxidation, in turn, enhances granule hydrophilicity and is consistent with the increased solubility and slight crystallinity reduction seen in PAW‐treated samples, as RONS preferentially target amorphous regions and crystal surfaces, initiating localized depolymerization and disrupting helical packing without complete crystal destruction (Gebremical et al. 2023). When PAW‐pretreated starches are subjected to autoclaving, the acidic environment and pre‐existing oxidative defects facilitate more extensive thermal hydrolysis. The literature confirms that PAW can induce both depolymerization and crosslinking depending on treatment conditions (Chou et al. 2023), and the presence of H^+^ in PAW promotes the depolymerization of amylopectin chains (Yan et al. 2025). Additionally, starch modification by PAW occurs through cross‐linking, plasma etching, and depolymerization mechanisms mediated by plasma species.

### 3.2 Hydrodynamic Radius

The hydrodynamic radius (Rh) of the amylose chains [Rh (am)] remained unchanged after individual treatments with plasma‐activated water (PAW) for 30 and 60 min (P30 and P60) (Figure 1A). However, a significant reduction was observed after autoclave treatment (AC), and this effect was drastically enhanced in the combined treatments that associated autoclaving with pre‐exposure to PAW (P30AC and P60AC). The same behavior was observed for the hydrodynamic radius of the starch as a whole [Rh (Whole)]; the chains were significantly reduced compared to the Native sample only after autoclaving, and the action of PAW intensified this treatment, demonstrating that the combined treatments were more effective at debranching the starch.

**FIGURE 1:**
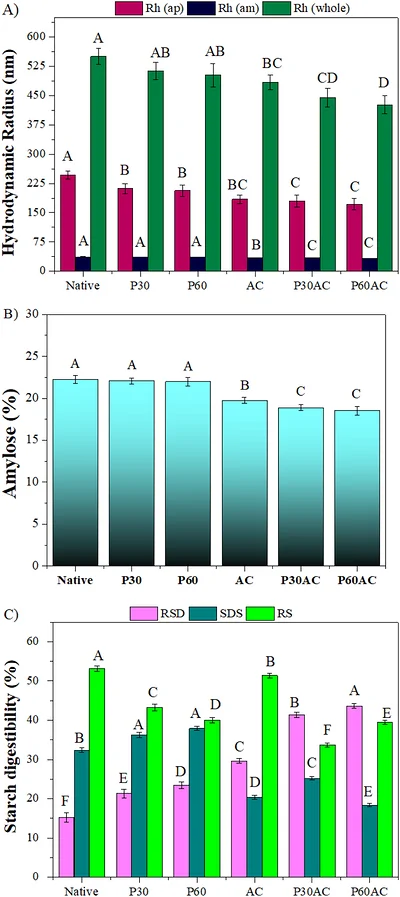
Physicochemical parameters of black rice starch: (A) hydrodynamic radius of amylose, amylopectin and whole, (B) Amylose content and (C) in vitro digestibility. Native, black rice native starch; P30, black rice starch hydrated with plasma‐activated water (PAW) for 30 min; P60, black rice starch hydrated with plasma‐activated water (PAW) for 60 min; AC, native starch suspended in distilled water and autoclaved (121°C, 3 min); P30AC, starch suspended in plasma‐activated water for 30 min and autoclaved for 3 min; P60AC, starch suspended in plasma‐activated water for 60 min and autoclaved for 3 min. Different uppercase letters above the bars indicate significant differences between treatments within the same fraction (*p* < 0.05).

Regarding the amylopectin chains [Rh (ap)], a distinct behavior was observed. While PAW treatment alone (P30 and P60) did not significantly alter Rh (am) and Rh (whole), it promoted a notable reduction in Rh (ap), suggesting that the reactive species generated in plasma preferentially attack the more exposed and branched external chains of amylopectin. This indicates that amylopectin, due to its highly branched and less compact structure, is more susceptible to the oxidative action of PAW than the more linear and densely packed amylose chains. Furthermore, the combined treatments (P30AC and P60AC) resulted in the most pronounced reductions in Rh (ap), confirming that the association of PAW and autoclaving is particularly effective in degrading the amylopectin fraction.

The stability of Rh (whole) and Rh (am) after the exclusive PAW treatments (P30 and P60) suggests that the reactive species generated in the plasma (such as free radicals, hydrogen peroxide, and ozone) were not, individually, capable of significantly degrading the long polymeric starch chains. Our findings suggest that PAW treatment alone does not provide sufficient energy for extensive glycosidic bond cleavage. This aligns with previous observations that plasma treatments often require combination with thermal processes to achieve significant starch structural alterations (Thirumdas et al. (2017).

On the other hand, the wet thermal treatment of AC alone induced a reduction in Rh (whole), Rh (am), and Rh (ap), an effect classically attributed to the thermal hydrolysis of amylose chains and the long external branches of amylopectin, as described by Varatharajan et al. (2011). Although statistically significant differences were observed between AC and the combined treatments (P30AC and P60AC) for Rh (am), the magnitude of these differences was relatively small, as the values were visually comparable. This suggests that the degree of depolymerization of amylose may not be of great practical relevance. Regarding the combined treatments, the reductions in Rh (ap) for P30AC and P60AC were less pronounced, indicating that extensive depolymerization of amylopectin did not occur. Nevertheless, the consistent trend of reduction across all Rh parameters indicates that the association of PAW pre‐treatment and autoclaving promoted greater structural modifications than either treatment alone, as confirmed by the pairwise comparisons (*p* < 0.05). It is plausible that the pre‐treatment with PAW causes a “pre‐weakening” of the starch granules, possibly through the oxidation of hydroxyl groups and surface erosion, which may facilitate subsequent structural modifications, as demonstrated by Thirumdas et al. (2016). However, this pre‐weakening appears to facilitate relatively mild structural modifications, rather than substantial chain cleavage.

The reduction in Rh values observed for the combined treatments correlates well with the chain length redistribution data (Section 3.4), as the decrease in hydrodynamic dimensions reflects the cleavage of long chains into shorter fragments. This is supported by the strong correlation between Rh (whole) and DP ≥37 (*r* = 0.91, *p* < 0.01), confirming that the reduction in molecular size is primarily due to depolymerization of long amylopectin chains.

### 3.3 Amylose Content

The results demonstrated that the apparent amylose content was significantly reduced only after the AC treatment, while exposure solely to plasma‐activated water (PAW) did not promote statistically relevant changes (*p* > 0.05) compared to native starch (Figure 1B). The most substantial depolymerization of amylose chains occurred under the combined treatments of PAW followed by autoclaving (P30AC and P60AC), reaching a maximum reduction of 16.76%. It was observed that although the application of PAW is a determining factor in enhancing subsequent thermal hydrolysis, the variation in water activation time (30 or 60 min) did not significantly influence the magnitude of the observed effect.

The absence of significant change in amylose content after the isolated PAW treatment corroborates the findings of Zhu (2017), who attributes this resistance to the physical barrier imposed by the organized and semi‐crystalline structure of the native starch granule, which protects the internal polymers from the action of short‐lived reactive species generated in the plasma. The reduction observed after the isolated AC treatment is a classic phenomenon of thermal hydrolysis, where moist heat under pressure promotes the breakdown of glycosidic bonds, resulting in smaller molecules and solubilization of amylose segments, thereby reducing the apparent content (Almeida et al. 2022).

The pronounced effect observed in the combined treatments (P30AC and P60AC) can be explained by a two‐step mechanism, supported by the literature. According to Taslikh et al. (2022), plasma can induce surface modifications, such as the formation of micropores and oxidation of hydroxyl groups, which “condition” the granule without, however, massively degrading the internal chains in isolation. This pre‐conditioning creates sites of weakness that are then intensively exploited during the subsequent thermal treatment. The pre‐treatment with PAW does not primarily act by breaking chains, but by modifying the granule architecture, facilitating the penetration of water vapor and heat during autoclaving. This exponentially amplifies thermal hydrolysis, leading to more extensive breakdown of amylose chains and consequently a greater reduction (Gupta et al. 2024).

### 3.4 Chain Length Distribution of Linear Glucans After Enzymatic Debranching

The fraction of shorter linear glucan chains (DP 6–12), derived from both amylose and amylopectin after enzymatic debranching, showed an initial reduction in the isolated PAW treatments (P30 and P60) compared to native starch, suggesting possible mild oxidative hydrolysis catalyzed by the reactive species present in PAW. However, an increase in this parameter was observed after the AC treatment and, more notably, in the combined treatments P30AC and P60AC. This is consistent with the fact that moist heat under pressure preferentially breaks glycosidic bonds, generating a larger number of oligosaccharides and short‐chain dextrins, as described by Xie et al. (2019).

Meanwhile, the fraction of intermediate‐length chains (DP 13–24) followed a similar increasing trend, while the long‐chain fractions (DP 25–36 and DP ≥37), representing longer linear glucan chains, underwent progressive and systematic reductions, especially in the combined treatments. This distribution profile evidences selective depolymerization, with preferential cleavage of longer chains, which are more susceptible to hydrolytic breakdown under heat and pressure (Chen et al. 2017). Although the magnitude of the changes in degree of polymerization was relatively small, the consistent trends observed across the chain length fractions indicate that the combined PAW and autoclaving treatments promoted a redistribution of linear glucan chains toward shorter fractions, which was more pronounced than in the isolated treatments for most parameters evaluated. This effect was more pronounced when PAW was applied for 60 min (P60AC), which may be attributed to the ability of plasma to erode the granule surface and create pores, thereby enhancing the accessibility of the subsequent hydrothermal treatment, as suggested by Ma and Jiang (2024).

PAW‐induced surface erosion may increase the accessibility of the granule interior to water and heat during autoclaving. This enhanced accessibility facilitates more efficient cleavage of glycosidic bonds in the amorphous regions and at the crystalline‐amorphous interfaces. As a result, long chains located in the more accessible outer layers and amorphous regions are preferentially hydrolyzed, generating shorter fragments that diffuse out of the granule or remain entrapped within the porous matrix. This mechanism is supported by the simultaneous increase in short‐chain fractions (DP 6–12) and reduction in long‐chain fractions (DP ≥ 37) observed in the combined treatments, which were more pronounced than in the AC treatment alone (*p* < 0.05).

The shift toward shorter chains observed in the combined treatments is consistent with the increased solubility (Section 3.6) and reduced pasting viscosities (Section 3.11), as shorter glucan chains are more hydrophilic and less able to form cohesive networks. This chain length redistribution also explains the improved freeze‐thaw stability (Section 3.10), since shorter chains have a lower tendency to retrograde.

### 3.5 In Vitro Digestibility

Native starch presented a digestibility profile characteristic of cereal starches (Figure 1C), with a considerable fraction of resistant starch (RS). This behavior is associated with the intact semi‐crystalline organization of the granule and the possible formation of complexes between amylose and phenolic compounds naturally present in black rice, which may restrict enzymatic access (Kraithong et al. 2024). Treatments with plasma‐activated water (P30 and P60) likely promoted superficial alterations on the granules, as reported by Gupta et al. (2024). These morphological changes tend to increase the contact area with digestive enzymes, which may result in a reduction of the RS fraction and a consequent increase in rapidly digestible starch (RDS), depending on the treatment intensity.

Autoclaving (AC) promotes starch gelatinization, disrupting the granular structure and disorganizing crystalline regions. This process inherently leads to a reduction in RS and an increase in RDS, consistent with the classic model of starch gelatinization proposed by Singh et al. (2003). According to Taslikh et al. (2022), prior structural modification of the granule surface via plasma‐activated water (PAW) facilitates more efficient and homogeneous gelatinization during autoclaving. This pre‐weakening allows heat and moisture to penetrate the granule more easily, promoting more complete molecular disorganization. Consequently, virtually all the starch becomes susceptible to immediate enzymatic hydrolysis, minimizing the RS fraction while maximizing RDS. Among the combined treatments, the lowest RS values were observed for P30AC (36.12%) and P60AC (25.73%), while the highest RDS values were recorded for P60AC (43.59%) and P30AC (41.36%). The slowly digestible starch (SDS) fractions for P30 (36.26%) and P60 (37.87%) showed no significant differences, indicating that PAW treatment alone did not substantially alter the intermediate digestibility fraction. The similarity between the results for P30AC and P60AC suggests that PAW exposure time may reach a saturation point, beyond which the facilitating effect on gelatinization is not significantly enhanced.

From a nutritional perspective, the increase in starch digestibility promoted by the combined treatments may be advantageous for certain applications. Although current food industry trends favor reducing starch digestibility to enhance dietary fiber content and mitigate postprandial glycemic responses, some products may benefit from starches with higher RDS and lower RS contents. Examples include formulations for rapid energy replenishment, sports nutrition, enteral diets, infant formulas, and convalescent products, where fast glucose availability is often required. Therefore, the proposed dual modification strategy offers a versatile approach for modulating starch digestibility according to the intended application, whether aiming for slow glucose release or rapid energy supply.

The increase in RDS and reduction in RS observed for the combined treatments are directly linked to the molecular depolymerization evidenced by the hydrodynamic radius reduction (Section 3.2) and the increase in short‐chain fractions (Section 3.4). The loss of crystalline order (Section 3.8) further enhances enzymatic accessibility, as the semi‐crystalline structure that normally restricts enzyme penetration is progressively disrupted.

### 3.6 Solubility of Starches

The low solubility exhibited by native starch (6.54%) is an intrinsic characteristic of its intact granular structure, organized into crystalline and amorphous regions, which drastically limits its interaction with water (Table 1). The isolated PAW treatments for 30 and 60 min promoted moderate and significant increases in solubility (10.09% and 13.36%, respectively). This initial increment can be attributed to the erosive effect of plasma, which increases the available surface area for hydration through surface modification and oxidation of hydroxyl groups into more hydrophilic carboxylic groups (‐COOH), as demonstrated by Okyere et al. (2022).

However, the most pronounced change was observed after the AC thermal treatment, which alone increased solubility to 22.12%. This is a result of the gelatinization process, in which moist heat under pressure disorganizes the crystalline structure, breaks internal hydrogen bonds, and promotes granular swelling, releasing soluble polymers. The maximum effect was achieved synergistically in the combined treatments P30AC and P60AC (29.57% and 31.57%, respectively). This increase in solubility is consistent with the changes in chain length distribution observed for these samples (Table 1), which showed a moderate increase in the short‐chain fraction (DP 6–12) and a reduction in the long‐chain fractions (DP ≥37). The correlation between these structural modifications and the enhanced solubility suggests that partial depolymerization of amylose chains and long branches of amylopectin contributes to the release of soluble polymers, although the magnitude of the chain length changes was relatively small (approximately 2% increase for DP 6–12 and 5.6% decrease for DP ≥37).

To further support the relationship between chain length redistribution and increased solubility, Pearson correlation analysis was performed between the chain length fractions (DP 6–12 and DP ≥37) and solubility values across all treatments. A strong positive correlation was observed between the short‐chain fraction (DP 6–12) and solubility (*r* = 0.97, *p* < 0.01), while a strong negative correlation was found between the long‐chain fraction (DP ≥37) and solubility (*r* = −0.98, *p* < 0.01). Additionally, DP 6–12 and DP ≥37 were strongly negatively correlated (*r* = −0.99, *p* < 0.01). These correlations confirm that the progressive increase in solubility observed with treatment severity is directly associated with the shift toward shorter glucan chains, supporting the mechanistic link between PAW‐induced depolymerization and enhanced starch hydration properties.

According to Ge et al. (2021), there is a fundamental correlation between the shortening of polymeric chains and the increase in solubility in starchy systems. The PAW pretreatment acts as a critical conditioning step that modifies the granule microstructure by oxidizing surface hydroxyl groups, introducing hydrophilic carboxyl moieties, and promoting localized surface erosion. These modifications weaken the structural integrity of the granule surface and amorphous regions, facilitating the penetration of heat and moisture during subsequent autoclaving, thereby enhancing thermal hydrolysis and gelatinization beyond what is achieved by autoclaving alone. This priming effect is supported by the increased carbonyl and carboxyl contents (Table 1) and the enhanced solubility observed for PAW‐treated samples, indicating that surface oxidation and structural weakening are key factors in the combined effect of PAW and autoclaving.

### 3.7 Morphology and Size of Starch Granules

Based on the average diameter values provided in Table 1 and Figure 2, significant morphological alterations were observed in black rice starch granules subjected to different treatments. Native starch granules exhibited typical morphological characteristics, with an average diameter of 6.89 µm. The granules maintained their structural integrity with intact surfaces, consistent with the features of unmodified starch (Almeida et al. 2023). After treatment with PAW for 30 min (P30), the diameter increased to 7.02 µm, although this value did not differ significantly from the native sample. The micrographs revealed slight surface swelling and surface erosion resulting from the oxidative action of plasma reactive species, along with surface erosion without complete structural disruption (Guo et al. 2021). In contrast, Shen et al. (2021) observed surface corrosion and increased damage with combined treatments (ultrasound + PAW), in addition to reduced swelling and greater thermal stability.

**FIGURE 2:**
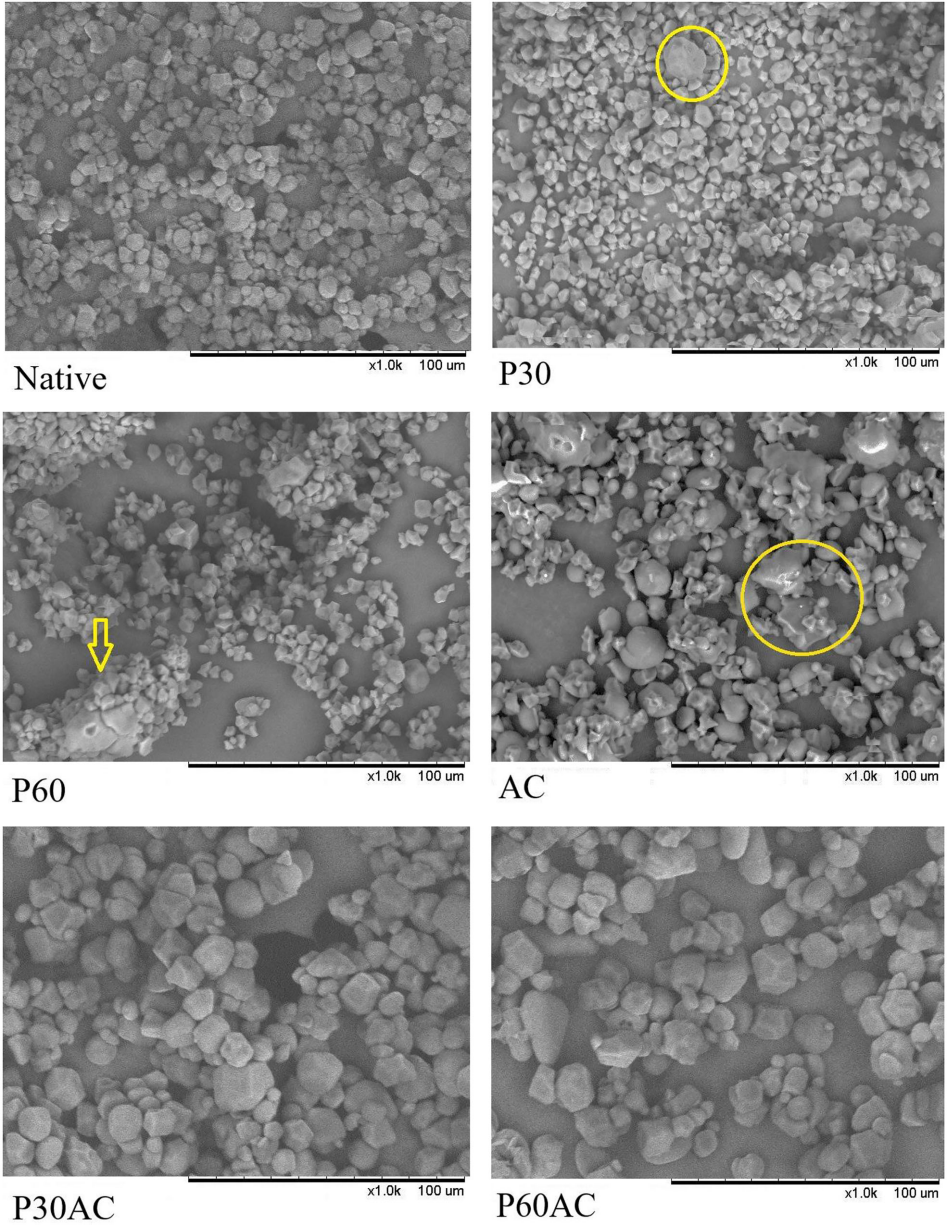
Surface and morphological parameters of starch: Native, black rice native starch; P30, black rice starch hydrated with plasma‐activated water (PAW) for 30 min; P60, black rice starch hydrated with plasma‐activated water (PAW) for 60 min; AC, native starch suspended in distilled water and autoclaved (121°C, 3 min); P30AC, starch suspended in plasma‐activated water for 30 min and autoclaved for 3 min; P60AC, starch suspended in plasma‐activated water for 60 min and autoclaved for 3 min.

The P60 treatment (PAW for 60 min) resulted in a diameter of 8.43 µm, with more pronounced and significant erosion, evident cracking, and partial aggregation, indicating that prolonged PAW exposure intensifies surface degradation, facilitating water absorption and swelling. The AC treatment showed a diameter of 10.06 µm, with disintegrated and aggregated granules, due to gelatinization, which disrupts molecular organization and promotes granule fragmentation and fusion (Guan et al. 2022).

The combined treatments P30AC and P60AC exhibited the largest diameters (11.27 µm and 12.50 µm, respectively) and were statistically different from each other, associated with severe morphological changes: more pronounced swelling, granule fusion, and formation of heterogeneous aggregates. The association of PAW and AC causes intense depolymerization, leading to the reorganization of polymers into larger and irregular structures, with partial loss of the original granular identity, as demonstrated by Sudheesh et al. (2019).

### 3.8 Crystalline Profile

The native starch exhibited a crystallinity of 25.18% (Table 1), a value characteristic of cereal starches with an A‐type X‐ray diffraction pattern, featuring characteristic peaks at approximately 15°, 17°, 18°, and 23° (2θ), as observed in Figure 3A and consistently reported in the literature for native starches (da Rosa Zavareze and Dias 2011).

**FIGURE 3:**
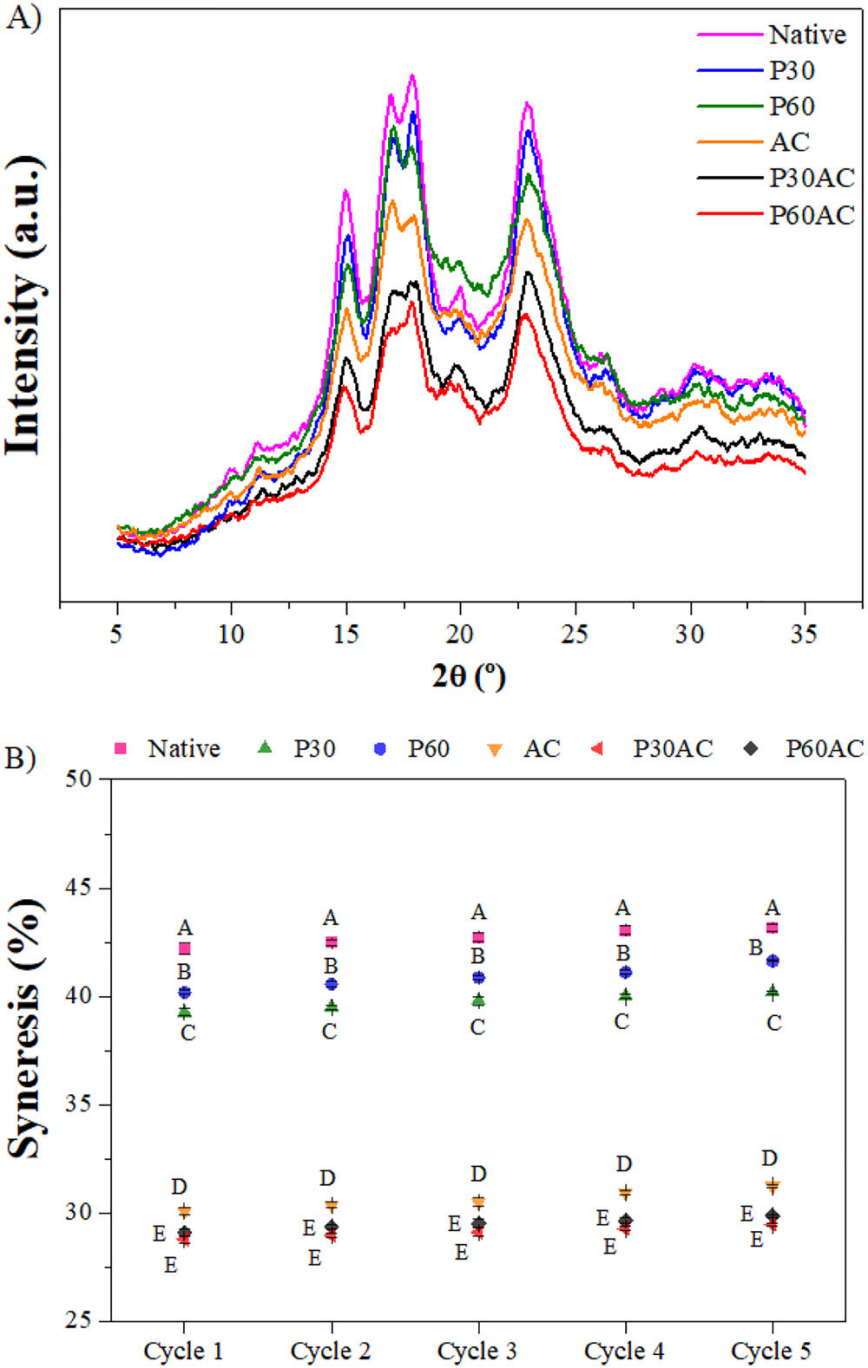
DRX (A) and Synereses (B): Native, black rice native starch; P30, black rice starch hydrated with plasma‐activated water (PAW) for 30 min; P60, black rice starch hydrated with plasma‐activated water (PAW) for 60 min; AC, native starch suspended in distilled water and autoclaved (121°C, 3 min); P30AC, starch suspended in plasma‐activated water for 30 min and autoclaved for 3 min; P60AC, starch suspended in plasma‐activated water for 60 min and autoclaved for 3 min. Different uppercase letters above the symbols indicate significant differences among treatments within the same freeze‐thaw cycle (*p* < 0.05).

Treatment with PAW for 30 min (P30) and 60 min (P60) promoted a significant yet moderate reduction in crystallinity (to 23.40% and 23.03%, respectively). The moderate crystallinity reduction after PAW treatment suggests surface and subsurface modification by RONS. These reactive species likely target amorphous regions and crystal surfaces, initiating localized depolymerization and disrupting helical packing without complete crystal destruction (Jiang et al., 2022). The similarity between the values for P30 and P60 suggests that exposure time was not effective in further reducing crystallinity.

The thermal treatment by AC resulted in a more pronounced reduction in crystallinity (21.81%). The hydrothermal treatment induced starch gelatinization, characterized by granular swelling and disruption of the crystalline domains. This structural disorganization, evidenced by XRD and DSC, reflects the conversion of the native semi‐crystalline architecture into a predominantly amorphous matrix (Watcharatewinkul et al. 2009). Although the hydrothermal conditions applied (121°C in excess water) are sufficient to induce gelatinization and partial disruption of the original crystalline order, the relatively short autoclaving time (3 min) may also limit the extent of crystallite disruption. This is because the high amylose content of black rice starch promotes rapid retrogradation during cooling. Furthermore, the presence of other cellular components within the starch matrix may partially restrict granule swelling and complete crystalline disorganization (Almeida et al. 2025).

The most pronounced effect was observed in the combined PAW + AC treatments (P30AC and P60AC), which resulted in the lowest crystallinities (20.57% and 19.68%, respectively). This reduction, confirmed by the pairwise comparisons (*p* < 0.05), demonstrates that the PAW pretreatment sensitizes the granule structure to the effects of subsequent heat. The plasma reactive species likely disorganize and weaken the surface and interior of the granules, creating “weak points” that facilitate more complete and efficient disruption during the autoclaving process (Ge et al. 2022). The moist heat can then penetrate and hydrolyze the crystalline regions that were previously “attacked” chemically by PAW more easily (Thirumdas et al. 2018). The fact that P60AC showed the lowest crystallinity value is consistent with the greater degradative action of prolonged PAW exposure, followed by thermal action, resulting in the highest structural disorganization.

The reduction in crystallinity observed for the combined treatments is consistent with the decreased gelatinization enthalpy (Section 3.9) and the increased solubility (Section 3.6), as the loss of ordered structure facilitates water penetration and molecular dissolution. The correlation between crystallinity and ΔH (*r* = 0.94, *p* < 0.01) confirms that the remaining crystalline regions are less stable and require less energy for disruption.

### 3.9 Thermal Parameters by DSC

The thermographic parameters (*T*_o_, *T*_c_, ΔH) reveal significant alterations in the thermal stability and molecular organization of black rice starch after the different treatments (Table 2). The native starch exhibits characteristic values of unmodified starches: gelatinization onset temperature (*T*_o_) of 68.18°C, conclusion temperature (*T*_c_) of 77.11°C, and enthalpy (ΔH) of 6.18 J·g^−1^, indicating an intact semi‐crystalline structure with stable double helices, as described by Ribeiro et al. (2023).

**TABLE 2:** Thermal and viscoamylographic properties of starch.

Parameters	Native	P30	P60	AC	P30AC	P60AC
***T*_o_ (°C)**	68.18 ± 0.29^B^	66.84 ± 0.32^C^	65.13 ± 0.15^E^	70.09 ± 0.48^A^	66.05 ± 0.14^D^	64.75 ± 0.21^F^
***T*_c_ (°C)**	77.11 ± 0.12^B^	76.74 ± 0.11^C^	76.29 ± 0.15^E^	78.86 ± 0.06^A^	76.22 ± 0.05^D^	75.88 ± 0.09^F^
**ΔH (J g^−1^)**	6.18 ± 0.04^B^	5.98 ± 0.09^C^	5.71 ± 0.04^E^	6.93 ± 0.15^A^	5.66 ± 0.09^D^	5.32 ± 0.08^F^
**PV (Pa s^−1^)**	19.40 ± 0.30^B^	20.66 ± 0.35^A^	10.97 ± 0.31^E^	16.40 ± 0.24^C^	15.86 ± 0.31^D^	9.47 ± 0.23^F^
**MV (Pa s^−1^)**	16.54 ± 0.32^A^	16.56 ± 0.21^A^	9.15 ± 0.12^D^	13.57 ± 0.63^B^	10.34 ± 0.23^C^	8.13 ± 0.12^E^
**BD (Pa s^−1^)**	2.86 ± 0.16^B^	4.11 ± 0.43^C^	1.82 ± 0.33^D^	2.83 ± 0.17^B^	5.52 ± 0.36^A^	1.34 ± 0.16^A^
**FV (Pa s^−1^)**	20.65 ± 0.27^B^	25.62 ± 0.44^A^	10.34 ± 0.29^D^	11.52 ± 0.34^C^	6.32 ± 0.22^F^	9.39 ± 0.14^E^

*Note*: Values are expressed (average ± standard deviation). Different superscript capital letters indicate that there is a significant difference (*p* < 0.05) between the formulations. Native, black rice native starch; P30, black rice starch hydrated with plasma‐activated water (PAW) for 30 min; P60, black rice starch hydrated with plasma‐activated water (PAW) for 60 min; AC, native starch suspended in distilled water and autoclaved (121°C, 3 min); P30AC, starch suspended in plasma‐activated water for 30 min and autoclaved for 3 min; P60AC, starch suspended in plasma‐activated water for 60 min and autoclaved for 3 min; *T*_o_, onset temperature; *T*_c_, completion temperature; ΔH, enthalpy of gelatinization; PV, maximum viscosity; MV, minimum viscosity; BD, breakdown; FV, final viscosity.

The isolated PAW treatments (P30 and P60) promote progressive reductions in *T*_o_, *T*_c_, and ΔH. In P60, *T*_o_ decreases to 65.13°C and ΔH to 5.71 J·g^−1^, suggesting that prolonged exposure to PAW causes surface erosion and disorganization of the crystalline structure, facilitating gelatinization with lower required energy (Zuo et al. 2024). The AC treatment results in an increase in *T*_o_ (70.09°C) and ΔH (6.93 J·g^−1^), which can be attributed to starch retrogradation and chain reassociation during the cooling phase following autoclaving. The hydrothermal treatment promotes gelatinization, disrupting the native crystalline structure; however, upon cooling, the released amylose chains and short amylopectin fragments reassociate into more ordered and thermally stable structures, such as type RS3 resistant starch (Ashwar et al. 2016; Faridah et al. 2022). This retrogradation process results in the formation of new crystalline regions with higher thermal stability, requiring greater energy (ΔH) to melt, as reflected by the increased *T*_o_ and ΔH values.

The combined treatments (P30AC and P60AC) show the lowest values of *T*_o_ (66.05°C and 64.75°C) and ΔH (5.66 J·g^−^^1^ and 5.32 J·g^−^^1^), indicating a combined effect between PAW and autoclaving. The preconditioning with PAW weakens the granule surface, allowing the autoclave to promote more extensive molecular disorganization, reducing thermal stability and the energy required for gelatinization, as elucidated by Aaliya et al. (2022). The consistent reduction in *T*_c_ in all treated samples, except AC, reflects lower stability of the remaining crystals.

### 3.10 Syneresis of the Paste

Native starch (Native) exhibited higher syneresis values than samples treated only with PAW (P30 and P60), which released minimal amounts of exuded water in each cycle (Figure 3B). This behavior is typical of native or mildly modified starch gels and can be attributed to two main factors. First, the presence of a high proportion of long amylose chains, which rapidly realign during cooling and storage, forming a rigid and contracted three‐dimensional network that expels water from the matrix. Second, the partially intact granular structure, even after gelatinization, provides contraction sites that favor water expulsion (Li et al., 2015). To support this interpretation, Pearson correlation analysis was performed between syneresis values and the structural parameters presented in Table 1. A strong positive correlation was observed between syneresis and the long‐chain fraction (DP ≥37) (*r* = 0.94, *p* < 0.01), while a strong negative correlation was found between syneresis and the short‐chain fraction (DP 6–12) (*r* = −0.89, *p* < 0.05). These correlations confirm that the higher syneresis observed in native starch is associated with the presence of longer glucan chains, which favor retrogradation and network contraction, whereas the reduction in syneresis in the modified samples is linked to the shift toward shorter chains.

In contrast, the combined treatments (P30AC and P60AC), together with AC alone, exhibited the lowest syneresis values when compared to the native starch, indicating superior gel stability during refrigerated storage. This reduction in water exudation is a direct consequence of the profound structural modifications induced by the synergy between PAW and autoclaving. The PAW pre‐treatment causes controlled surface erosion and the introduction of carboxyl groups on the granule surface (Zuo et al. 2024). This surface modification allows subsequent autoclaving to promote more efficient and homogeneous thermal hydrolysis, resulting in the selective depolymerization of long amylose chains and long external branches of amylopectin, as evidenced by the increase in the short‐chain fraction (DP 6–12) (Table 1). The generation of these lower molecular weight segments is the critical factor for the reduction in syneresis, since shorter chains have a lower tendency to retrograde and form rigid junction points within the gel network. Instead, they act as “spacers” or plasticizers, creating a more flexible polymer matrix with greater water retention capacity, even under cyclic stress (da Rosa Zavareze and Dias 2011).

The improved freeze‐thaw stability of the combined treatments can be attributed to the chain length redistribution (Section 3.4), as shorter glucan chains act as plasticizers and reduce the tendency for amylose retrogradation and network contraction. This is consistent with the reduced pasting viscosities (Section 3.11), indicating that the modified starches have a lower capacity to form rigid gel networks.

### 3.11 RVA Analysis of Starches

The rheological analysis of black rice starch demonstrates a clear dependence of the effects of plasma‐activated water (PAW) treatment on exposure time and its combined effect with thermal treatment (Table 2). The peak viscosity (PV), which reflects the granule swelling capacity, increased from 19.40 Pa·s^−^^1^ (native) to 20.66 Pa·s^−^^1^ after 30 min of PAW (P30), suggesting that shorter exposure superficially modifies the granule, facilitating greater hydration and swelling (Jiang et al., 2022). In contrast, prolonged exposure of 60 min (P60) resulted in a drastic reduction in PV (10.97 Pa·s^−^^1^) and minimum viscosity (MV; 9.15 Pa·s^−^^1^), indicating molecular degradation due to oxidation and chain breakdown, as reported in plasma treatments (Zhu 2017). The contrasting behavior between P30 and P60 can be explained by the extent of surface modification induced by PAW. At shorter exposure times (30 min), the reactive species primarily oxidize the granule surface, increasing hydrophilicity and promoting water absorption without extensive chain scission, which enhances swelling capacity. However, prolonged exposure (60 min) allows deeper penetration of reactive species, leading to more extensive chain cleavage, as evidenced by the increased short‐chain fraction (DP 6–12) (Table 1) and the reduced long‐chain fractions (DP ≥37) observed for P60. This molecular degradation weakens the granular integrity, limiting the swelling capacity during heating and resulting in lower PV and MV (Zhu 2017; Zuo et al. 2024).

The isolated thermal treatment (AC) also moderately reduced PV (16.40 Pa·s^−^^1^) and MV (13.57 Pa·s^−^^1^), due to gelatinization and partial granule rupture (Watcharatewinkul et al. 2009). However, the combined PAW + AC treatments (P30AC and P60AC) showed a more pronounced degradation effect, with P60AC recording the lowest values of PV (9.47 Pa·s^−1^) and MV (8.13 Pa·s^−1^), evidencing that the PAW pretreatment sensitizes the starch to thermal disintegration (Zuo et al. 2024).

The breakdown (BD) parameter, an indicator of paste stability, corroborates this interpretation. P30AC exhibited the highest BD (5.52 Pa·s^−^^1^), revealing a swollen but unstable gelatinized structure susceptible to shear (Grgić et al. 2019). In contrast, the P60, AC, and P60AC treatments showed very low BD values. The breakdown (BD) parameter, defined as the difference between PV and MV, is often used as an indicator of paste stability under shear and heat. P30AC exhibited the highest BD (5.52 Pa·s^−^^1^), indicating that despite having a moderately reduced PV, the granules swelled sufficiently during heating but the resulting paste was unstable and susceptible to shear‐induced disruption (Grgić et al. 2019). In contrast, the P60, AC, and P60AC treatments showed very low BD values, which is consistent with their already reduced PV and MV. Since the peak viscosity was already low, there was little swollen granular structure available to be broken down during the holding phase, resulting in minimal differences between PV and MV (Thirumdas et al. 2018).

The final viscosity (FV), related to gel formation, was highest in P30 (25.62 Pa·s^−^^1^), suggesting greater release and reassociation of amylose (Chen et al. 2017). However, the combined treatments P30AC (6.32 Pa·s^−^^1^) and P60AC (9.39 Pa·s^−^^1^) resulted in the lowest FVs, demonstrating that the PAW + heat synergy breaks the polymeric chains into excessively small fragments, incapable of reassociating and forming a gelled network—an advantageous effect for applications requiring reduced retrogradation (Zhang et al. 2022).

### 3.12 Pearson Correlation Analysis and Structure–Function Relationships

Pearson correlation analysis revealed that the enzymatic susceptibility of black rice starches is strongly governed by the structural order and molecular composition of the granules (Figure 4). Rapidly digestible starch (RDS) correlated negatively with amylose content (*r* = −0.961, *p* < 0.01) and crystallinity (*r* = −0.983, *p* < 0.001), while showing a strong positive association with solubility (*r* = 0.990, *p* < 0.001), indicating that disorganization of crystalline packing and the amorphous fraction facilitates cleavage by digestive enzymes. In contrast, slowly digestible starch (SDS) depended positively on amylose content (*r* = 0.904, *p* < 0.05) and on intermediate proportions of amylopectin chains (DP 25–36: *r* = 0.950, *p* < 0.01), confirming that the presence of reorganized double helices retards the action of the enzyme complex. Furthermore, long amylopectin chains (DP ≥37) and amylose content showed a direct positive correlation with crystallinity (*r* = 0.966, *p* < 0.01), acting to stabilize the crystallites. In the thermal and rheological profile, the gelatinization parameters (*T*_o_, *T*_c_, and ΔH) exhibited strong mutual interdependence (*r* = 0.950, *p* < 0.01), while minimum viscosity (MV) and final viscosity (FV) correlated positively (*r* = 0.868, *p* < 0.05), reflecting the capacity for reassociation of leached chains and starch retrogradation during cooling. These correlations are consistent with those observed between the short‐chain fraction (DP 6–12) and solubility (*r* = 0.97, *p* < 0.01), and between the long‐chain fraction (DP ≥37) and solubility (*r* = −0.98, *p* < 0.01), confirming that the shortening of polymeric chains is a determining factor for the functional properties of modified starch.

**FIGURE 4:**
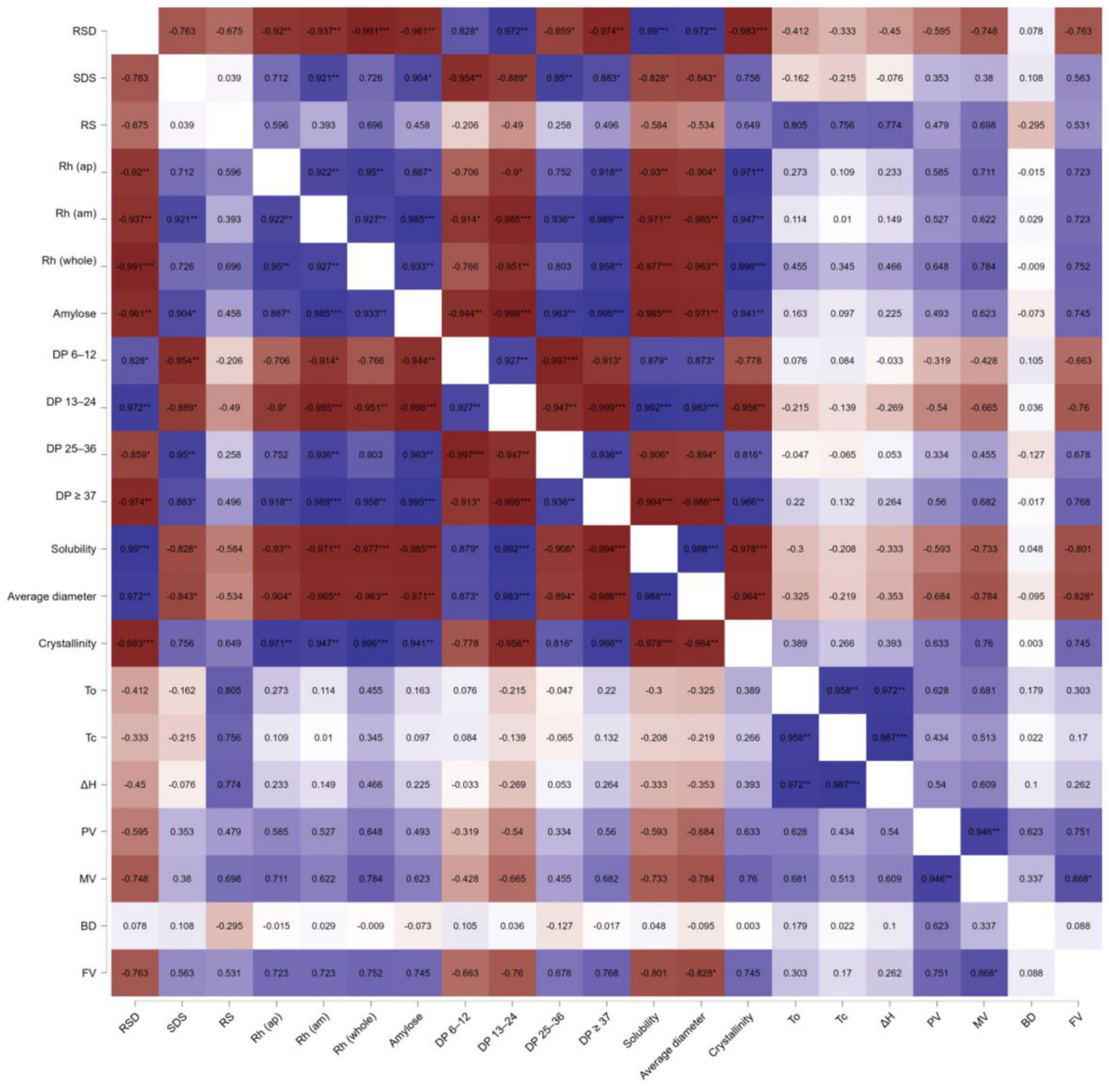
Pearson correlation matrix among physicochemical, morphological, structural, in vitro digestibility, thermal, and viscoamylographic properties of native and modified black rice starches. *p < 0.05, **p < 0.01, ***p < 0.001. RDS, rapidly digestible starch; SDS, slowly digestible starch; RS, resistant starch; *T*_o_, onset temperature; *T*_c_, completion temperature; ΔH, enthalpy of gelatinization, PV, peak viscosity; MV, minimum viscosity; BD, breakdown; FV, final viscosity.

## 4 Conclusions

The structural transformations induced by the dual modification were evidenced by various complementary analyses. Chemical analysis confirmed that PAW induced progressive surface oxidation of starch granules, as evidenced by increased carbonyl and carboxyl contents, reaching maximum values of 1.12 C = O/100 g glucose and 0.68 COOH/100 g glucose for P60AC. The hydrodynamic radius (Rh) of amylose and amylopectin chains was significantly reduced, accompanied by a shift in the distribution of linear glucan chains toward shorter fractions (DP 6–12) after enzymatic debranching, and a decrease in apparent amylose content. These molecular alterations translated into pronounced changes at the granular level, including progressive swelling (diameter increase from 6.89 to 12.50 µm), reduced relative crystallinity (from 25.18% to 19.68%), and increased solubility (up to 31.57%).

Thermal analysis confirmed that the remaining crystalline regions required less energy for disruption (ΔH = 5.32 J g^−^^1^), while pasting properties were drastically reduced, indicating extensive molecular degradation. From a functional perspective, the combined treatments resulted in higher starch digestibility, characterized by an increase in rapidly digestible starch (RDS) content and a reduction in the resistant starch (RS) fraction; this can be advantageous for applications requiring rapid glucose release, such as sports nutrition, enteral formulations, and infant foods. Furthermore, the reduced syneresis values observed in the combined treatments indicate improved freeze‐thaw stability, attributed to the generation of shorter glucan chains that act as plasticizers and reduce amylose retrogradation and network contraction.

This sequential approach represents a promising strategy for targeted starch modification, wherein non‐thermal plasma pre‐conditioning enhances subsequent thermal effects, yielding outcomes that surpass merely additive interactions. The study confirms that PAW pre‐treatment effectively sensitizes starch granules to thermal degradation, enabling more efficient and controllable modification.

## Author Contributions

**Raphael Lucas Jacinto Almeida**: methodology, conceptualization, writing – original draft. **Newton Carlos Santos**: methodology. **João Vitor Fonseca Feitoza**: writing – original draft. **Iêda Letícia de Souza Ferreira**: validation, visualization. **Raphael da Silva Eduardo**: methodology. **André Miranda da Silva**: investigation. **Cecilia Elisa Sousa Muniz**: visualization. **Ellen Cristina Nabiça Rodrigues**: supervision, project administration. **Alison Bruno Borges de Sousa**: supervision. **Lícia Amazonas Calandrini Braga**: funding acquisition. **Adriano Cesar Calandrini Braga**: funding acquisition. **Victor Herbert de Alcântara Ribeiro**: data curation. **Ynis Thaciane Felix da Silva**: data curation. **Martha Maria Gomes Nóbrega**: resources. **Ana Carla Oliveira de Brito**: resources, visualization. **Virgínia Mirtes de Alcantara Silva**: supervision.

## Funding

Newton Santos were supported by National Council for Scientific and Technological Development.

## Ethics Statement

This study did not involve human participants, animal subjects, or any data requiring ethical oversight by an institutional review board. Therefore, ethical approval was not sought.

## Conflicts of Interest

The authors declare that there are no financial, professional, or personal conflicts of interest that could have influenced the work reported in this paper.

## Data Availability

The datasets generated and analyzed during the current study are not publicly available due to ongoing research, but can be made available by the corresponding author upon justified and reasonable request.

## References

[jfds71534-bib-0001] Aaliya, B. , K. Sunooj , M. Navaf , et al. 2022. “Influence of Plasma‐Activated Water on the Morphological, Functional, and Digestibility Characteristics of Hydrothermally Modified Non‐Conventional Talipot Starch.” Food Hydrocolloids 130: 107709. 10.1016/j.foodhyd.2022.107709.

[jfds71534-bib-0002] Almeida, R. L. J. , N. S. Rios , and E. S. Dos Santos . 2022. “Modification of Red Rice Starch by a Combination of Hydrothermal Pretreatments and α‐Amylase Hydrolysis.” Carbohydrate Polymers 296: 119963. 10.1016/j.carbpol.2022.119963. 36088003

[jfds71534-bib-0003] Almeida, R. L. J. , N. C. Santos , W. R. D. B. de Medeiros , A. P. R. de Queiroga , N. S. Rios , and E. S. dos Santos . 2025. “Dual Modification of Red Rice Starch: Combining Hydrothermal Pretreatment With Acid Hydrolysis for the Production of Nanomaterials.” Chemical Engineering and Processing—Process Intensification 213: 110292. 10.1016/j.cep.2025.110292.

[jfds71534-bib-0004] Almeida, R. L. J. , N. C. Santos , J. V. F. Feitoza , et al. 2023. “Influence of Annealing Process Combined With Enzymatic Modification With Transglucosidase on Black Rice Starch.” Starch—Stärke 75, no. 3‐4: 2200161. 10.1002/star.202200161.

[jfds71534-bib-0005] Almeida, R. L. J. , N. C. Santos , J. V. F. Feitoza , et al. 2024. “Evaluation of Dual Modification by High Hydrostatic Pressure and Annealing on the Physicochemical Properties of Bean Starch.” Food Research International 177: 113877. 10.1016/j.foodres.2023.113877. 38225140

[jfds71534-bib-0006] Almeida, R. L. J. , N. C. Santos , C. E. Padilha , S. S. Monteiro , and E. S. Santos . 2021. “Impact of Hydrothermal Pretreatments on Physicochemical Characteristics and Drying Kinetics of Starch From Red Rice (Oryza sativa L.).” Journal of Food Processing and Preservation 45, no. 5: e15448. 10.1111/jfpp.15448.

[jfds71534-bib-0054] Almeida, R. L. J. , Dos Santos Pereira, T. , De Andrade Freire, V. , et al. 2019. “Influence of Enzymatic Hydrolysis on the Properties of Red Rice Starch.” International Journal of Biological Macromolecules 141: 1210–1219. 10.1016/j.ijbiomac.2019.09.072. 31521658

[jfds71534-bib-0007] An, J. , Z. Zhang , and Y. Yao . 2025. “Optimizing Processing Sequences in Combined Techniques for Rice Starch Modification: A Mini Review of Current Understanding.” Journal of Cereal Science 123: 104179. 10.1016/j.jcs.2025.104179.

[jfds71534-bib-0008] Ashwar, B. A. , A. Gani , I. A. Wani , A. Shah , F. A. Masoodi , and D. C. Saxena . 2016. “Production of Resistant Starch From Rice by Dual Autoclaving‐Retrogradation Treatment: In Vitro Digestibility, Thermal and Structural Characterization.” Food Hydrocolloids 56: 108–117. 10.1016/j.foodhyd.2015.12.004.

[jfds71534-bib-0009] Bai, T.‐G. , L. Zhang , J.‐Y. Qian , et al. 2021. “Pulsed Electric Field Pretreatment Modifying Digestion, Texture, Structure and Flavor of Rice.” LWT 138: 110650. 10.1016/j.lwt.2020.110650.

[jfds71534-bib-0010] Bhat, F. M. , S. R. Sommano , C. S. Riar , P. Seesuriyachan , T. Chaiyaso , and C. Prom‐u‐Thai . 2020. “Status of Bioactive Compounds From Bran of Pigmented Traditional Rice Varieties and Their Scope in Production of Medicinal Food With Nutraceutical Importance.” Agronomy 10: 1817. 10.3390/agronomy10111817.

[jfds71534-bib-0011] Chen, Y. , Q. Yang , X. Xu , et al. 2017. “Structural Changes of Waxy and Normal Maize Starches Modified by Heat Moisture Treatment and Their Relationship With Starch Digestibility.” Carbohydrate Polymers 177: 232–240. 10.1016/j.carbpol.2017.08.121 28962764

[jfds71534-bib-0012] Chou, Y. J. , Y. H. Tseng , K. C. Hsieh , and Y. Ting . 2023. “The Effect of Plasma‐Activated Water Treatment on the Physicochemical Properties of Potato Starch.” Food Bioscience 53: 102613. 10.1016/j.fbio.2023.102613.

[jfds71534-bib-0014] Englyst, H. N. , S. M. Kingman , and J. H. Cummings . 1992. “Classification and Measurement of Nutritionally Important Starch Fractions.” European Journal of Clinical Nutrition 46: S33–50. 1330528

[jfds71534-bib-0015] Faridah, D. , R. Silitonga , D. Indrasti , F. Afandi , A. Jayanegara , and M. Anugerah . 2022. “Verification of Autoclaving‐Cooling Treatment to Increase the Resistant Starch Contents in Food Starches Based on Meta‐Analysis Result.” Frontiers in Nutrition 9: 904700. 10.3389/fnut.2022.904700. PMC9343710 35928838

[jfds71534-bib-0016] Ge, X. , Y. Guo , J. Zhao , J. Zhao , H. Shen , and W. Yan . 2022. “Dielectric Barrier Discharge Cold Plasma Combined With Cross‐linking: an Innovative Way to Modify the Multi‐scale Structure and Physicochemical Properties of Corn Starch.” International Journal of Biological Macromolecules 215: 465–476. 10.1016/j.ijbiomac.2022.06.060 35714867

[jfds71534-bib-0017] Ge, X. , H. Shen , C. Su , et al. 2021. “The Improving Effects of Cold Plasma on Multi‐Scale Structure, Physicochemical and Digestive Properties of Dry Heated Red Adzuki Bean Starch.” Food Chemistry 349: 129159. 10.1016/j.foodchem.2021.129159. 33545604

[jfds71534-bib-0018] Gebremical, G. G. , S. Tappi , R. Laurita , et al. 2023. “Effects of Plasma Activated Water (PAW) on Rheological, Thermal, Hydration and Pasting Properties of Normal Maize, Waxy Maize and Potato Starches.” Food Hydrocolloids 144: 109006. 10.1016/j.foodhyd.2023.109006.

[jfds71534-bib-0019] Grgić, I. , Đ. Ačkar , V. Barišić , M. Vlainić , N. Knežević , and Z. Medverec Knežević . 2019. “Nonthermal Methods for Starch Modification—A Review.” Journal of Food Processing and Preservation 43, no. 12: e14242. 10.1111/jfpp.14242.

[jfds71534-bib-0020] Guan, Y. , M. Wang , X. Song , et al. 2022. “Study on Structural Characteristics, Physicochemical Properties, and In Vitro Digestibility of Kudzu‐Resistant Starch Prepared by Different Methods.” Food Science & Nutrition 11: 481–492. 10.1002/fsn3.3079. PMC9834852 36655107

[jfds71534-bib-0021] Guo, Z. , Q. Gou , L. Yang , Q. Yu , and L. Han . 2021. “Dielectric Barrier Discharge Plasma: a Green Method to Change Structure of Potato Starch and Improve Physicochemical Properties of Potato Starch Films.” Food Chemistry 370: 130992. 10.1016/j.foodchem.2021.130992. 34509946

[jfds71534-bib-0055] Guo, P. , Yu, J. , Copeland, L. , Wang, S. , and Wang, S. 2018. “Mechanisms of Starch Gelatinization During Heating of Wheat Flour and Its Effect on in Vitro Starch Digestibility.” Food Hydrocolloids 82: 370–378. 10.1016/j.foodhyd.2018.04.012.

[jfds71534-bib-0022] Gupta, R. K. , P. Guha , and P. P. Srivastav . 2024. “Effect of Cold Plasma Treatment and Plasma‐Activated Water on Physicochemical and Structural Properties of Starch: a Green and Novel Approach for Environmental Sustainability.” Plasma Processes and Polymers 21, no. 4: 2300204. 10.1002/ppap.202300204.

[jfds71534-bib-0056] Jane, J. L. , Chen, Y. Y. , Lee, L. F. , et al. 1999. “Effects of Amylopectin Branch Chain Length and Amylose Content on the Gelatinization and Pasting Properties of Starch.” Cereal Chemistry 76, no. 5: 629–637. 10.1094/cchem.1999.76.5.629.

[jfds71534-bib-0057] Jiang, F. , Du, C. , Zhao, N. , Jiang, W. , Yu, X. , and Du, S. K. 2022. “Preparation and Characterization of Quinoa Starch Nanoparticles as Quercetin Carriers.” Food Chemistry 369: 130895. 10.1016/j.foodchem.2021.130895. 34438343

[jfds71534-bib-0025] Junden, S. , A. Panya , M. Umar , S. Raungrusmee , and A. K. Anal . 2025. “Improving the Techno‐Functional Properties and Glycemic Index of Khai Mod Rin Rice Starch Through Autoclaving–Chilling Cycles.” International Journal of Biological Macromolecules 315: 144470. 10.1016/j.ijbiomac.2025.144470. 40414392

[jfds71534-bib-0026] Kraithong, S. , A. Theppawong , N. Bunyameen , X. Zhang , and R. Huang . 2024. “Advancements in Understanding Low Starch Hydrolysis in Pigmented Rice: A Comprehensive Overview of Mechanisms.” Food Chemistry 439: 138079. 10.1016/j.foodchem.2023.138079. 38043273

[jfds71534-bib-0027] Kumari, B. , and N. Sit . 2023. “Comprehensive Review on Single and Dual Modification of Starch: Methods, Properties and Applications.” International Journal of Biological Macromolecules 253: 126952. 10.1016/j.ijbiomac.2023.126952. 37722643

[jfds71534-bib-0058] Leach, H. W. 1959. “Structure of Starch Granule I. Swelling and Solubility Patterns of Various Starches.” J. Cereal Chem. 36: 534–544.

[jfds71534-bib-0059] Li, W. , Tian, X. , Liu, L. , et al. 2015. “High Pressure Induced Gelatinization of Red Adzuki Bean Starch and Its Effects on Starch Physicochemical and Structural Properties.” Food Hydrocolloids 45: 132–139. 10.1016/j.foodhyd.2014.11.013.

[jfds71534-bib-0060] Lima, D. C. , Villar, J. , Castanha, N. , Maniglia, B. C. , Junior, M. D. M. , and Augusto, P. E. D. 2020. “Ozone Modification of Arracacha Starch: Effect on Structure and Functional Properties.” Food Hydrocolloids 108: 106066. 10.1016/j.foodhyd.2020.106066.

[jfds71534-bib-0028] Lin, J. , E. Li , and C. Li . 2024. “Multi‐Scale Structural Insights on Starch Digestibility of Instant Rice.” Food Chemistry 456: 140074. 10.1016/j.foodchem.2024.140074. 38876074

[jfds71534-bib-0030] Liu, H. , G. Tian , S. Gao , and S. Liu . 2026. “Understanding the Changes in Multi‐Structure, Physicochemical Properties, and In Vitro Digestibility of Quinoa Starch by Annealing With Hydrogen‐infused and Plasma‐Activated Water Samples.” Journal of Future Foods 6, no. 2: 292–300. 10.1016/j.jfutfo.2024.04.012.

[jfds71534-bib-0031] Ma, S. , and H. Jiang . 2024. “The Effect of Cold Plasma on Starch: Structure and Performance.” Carbohydrate Polymers 340: 122254. 10.1016/j.carbpol.2024.122254. 38857998

[jfds71534-bib-0032] Miller, G. L. 1959. “Use of Dinitrosalicylic Acid Reagent for Determination of Reducing Sugar.” Analytical Chemistry 31, no. 3: 426–428. 10.1021/ac60147a030.

[jfds71534-bib-0061] Nadiha, M. N. , Fazilah, A. , Bhat, R. , and Karim, A. A. 2010. “Comparative Susceptibilities of Sago, Potato and Corn Starches to Alkali Treatment.” Food Chemistry 121, no. 4: 1053–1059. 10.1016/j.foodchem.2010.01.048.

[jfds71534-bib-0033] Okyere, A. , P. Boakye , E. Bertoft , and G. Annor . 2022. “Temperature of Plasma‐Activated Water and Its Effect on the Thermal and Chemical Surface Properties of Cereal and Tuber Starches.” Current Research in Food Science 5: 1668–1675. 10.1016/j.crfs.2022.09.020. PMC9526130 36193040

[jfds71534-bib-0034] Ribeiro, V. H. D. A. , M. E. R. M. Cavalcanti‐Mata , R. L. J. Almeida , and V. M. D. A. Silva . 2023. “Characterization and Evaluation of Heat–Moisture‐Modified Black and Red Rice Starch: Physicochemical, Microstructural, and Functional Properties.” Foods 12, no. 23: 4222. 10.3390/foods12234222. PMC10706738 38231608

[jfds71534-bib-0013] da Rosa Zavareze, E. , and A. R. G. Dias . 2011. “Impact of Heat‐Moisture Treatment and Annealing in Starches: A Review.” Carbohydrate Polymers 83, no. 2: 317–328. 10.1016/j.carbpol.2010.08.064

[jfds71534-bib-0035] Shen, H. , Y. Guo , J. Zhao , et al. 2021. “The Multi‐Scale Structure and Physicochemical Properties of Mung Bean Starch Modified by Ultrasound Combined With Plasma Treatment.” International Journal of Biological Macromolecules 191: 821–831. 10.1016/j.ijbiomac.2021.09.157. 34597694

[jfds71534-bib-0036] Sifuentes‐Nieves, I. , A. Soler , and P. C. Flores‐Silva . 2024. “Effect of Plasma‐activated Water on the Supramolecular Structure and Techno‐Functional Properties of Starch: A Review.” Food Chemistry 456: 139997. 10.1016/j.foodchem.2024.139997. 38865820

[jfds71534-bib-0037] Singh, N. , J. Singh , L. Kaur , N. Singh Sodhi , and B. Singh Gill . 2003. “Morphological, Thermal and Rheological Properties of Starches From Different Botanical Sources.” Food Chemistry 81, no. 2: 219–231. 10.1016/S0308-8146(02)00416-8.

[jfds71534-bib-0038] Sudheesh, C. , K. V. Sunooj , S. K. Sinha , et al. 2019. “Impact of Energetic Neutral Nitrogen Atoms Created by Glow Discharge Air Plasma on the Physico‐Chemical and Rheological Properties of Kithul Starch.” Food chemistry 294: 194–202. 10.1016/j.foodchem.2019.05.067. 31126453

[jfds71534-bib-0062] Syahariza, Z. A. , Sar, S. , Hasjim, J. , Tizzotti, M. J. , and Gilbert, R. G. 2013. “The Importance of Amylose and Amylopectin Fine Structures for Starch Digestibility in Cooked Rice Grains.” Food Chemistry 136, no. 2: 742–749. 10.1016/j.foodchem.2012.08.053. 23122122

[jfds71534-bib-0039] Taslikh, M. , H. Abbasi , A. M. Mortazavian , J. B. Ghasemi , A. Naeimabadi , and K. Nayebzadeh . 2022. “Effect of Cold Plasma Treatment, Cross‐Linking, and Dual Modification on Corn Starch.” Starch—Stärke 74, no. 5‐6: 2200008. 10.1002/star.202200008.

[jfds71534-bib-0040] Thirumdas, R. , D. Kadam , and U. S. Annapure . 2017. “Cold Plasma: An Alternative Technology for the Starch Modification.” Food Biophysics 12, no. 1: 129–139. 10.1007/s11483-017-9468-5.

[jfds71534-bib-0041] Thirumdas, R. , A. Kothakota , U. Annapure , et al. 2018. “Plasma Activated Water (PAW): Chemistry, Physico‐chemical Properties, Applications in Food and Agriculture.” Trends in Food Science & Technology 77: 21–31. 10.1016/j.tifs.2018.05.007.

[jfds71534-bib-0042] Thirumdas, R. , C. Saragapani , M. T. Ajinkya , R. R. Deshmukh , and U. S. Annapure . 2016. “Influence of Low Pressure Cold Plasma on Cooking and Textural Properties of Brown Rice.” Innovative Food Science & Emerging Technologies 37: 53–60. 10.1016/j.ifset.2016.08.009.

[jfds71534-bib-0043] Varatharajan, V. , R. Hoover , J. Li , et al. 2011. “Impact of Structural Changes Due to Heat‐moisture Treatment at Different Temperatures on the Susceptibility of Normal and Waxy Potato Starches towards Hydrolysis by Porcine Pancreatic Alpha Amylase.” Food Research International 44, no. 9: 2594–2606. 10.1016/j.foodres.2011.04.050.

[jfds71534-bib-0044] Wang, J. , X. Xue , S. Han , et al. 2025. “Effects of Dual Modification by Cold Plasma and Heat‐moisture Treatment on the Structure and in Vitro Digestibility of Waxy Maize Starch.” Innovative Food Science & Emerging Technologies 102: 103991. 10.1016/j.ifset.2025.103991.

[jfds71534-bib-0045] Watcharatewinkul, Y. , C. Puttanlek , V. Rungsardthong , and D. Uttapap . 2009. “Pasting Properties of a Heat‐moisture Treated Canna Starch in Relation to Its Structural Characteristics.” Carbohydrate Polymers 75, no. 3: 505–511. 10.1016/j.carbpol.2008.08.018.

[jfds71534-bib-0046] Xie, Y. , M. Li , H. Chen , and B. Zhang . 2019. “Effects of the Combination of Repeated Heat‐moisture Treatment and Compound Enzymes Hydrolysis on the Structural and Physicochemical Properties of Porous Wheat Starch.” Food Chemistry 274: 351–359. 10.1016/j.foodchem.2018.09.034. 30372951

[jfds71534-bib-0047] Yan, Y. , J. Fang , X. Zhang , X. Ji , M. Shi , and B. Niu . 2025. “Insight Into Formation and Structure of Wheat Starch‐Lauric Acid Complexes by Extrusion: Effect of Plasma‐Activated Water.” Food Chemistry 469: 142640. 10.1016/j.foodchem.2024.142640. 39733567

[jfds71534-bib-0048] Yan, Y. , L. Feng , M. Shi , C. Cui , and Y. Liu . 2020. “Effect of Plasma‐activated Water on the Structure and in Vitro Digestibility of Waxy and Normal Maize Starches During Heat‐Moisture Treatment.” Food Chemistry 306: 125589. 10.1016/j.foodchem.2019.125589. 31606629

[jfds71534-bib-0049] Yan, Y. , X. Xue , X. Jin , et al. 2022. “Effect of Annealing Using Plasma‐activated Water on the Structure and Properties of Wheat Flour.” Frontiers in Nutrition 9: 951588. 10.3389/fnut.2022.951588. PMC9403792 36034897

[jfds71534-bib-0050] Zhang, K. , Z. Zhang , M. Zhao , et al. 2022. “Low‐Pressure Plasma Modification of the Rheological Properties of Tapioca Starch.” Food Hydrocolloids 125: 107380. 10.1016/j.foodhyd.2021.107380.

[jfds71534-bib-0051] Zhang, M. , Z. Xu , Q. Zhang , Z. Dan , H. Fu , and W. Yao . 2023. “Properties and Potential Application of Ozone‐Oxidized Starch for Enhanced Reverse Flotation of Fine Hematite.” Minerals Engineering 198: 108084. 10.1016/j.mineng.2023.108084.

[jfds71534-bib-0052] Zhu, F. 2017. “Plasma Modification of Starch.” Food Chemistry 232: 476–486. 10.1016/j.foodchem.2017.04.024. 28490100

[jfds71534-bib-0063] Zhu, D. , Zhang, H. , Guo, B. , Xu, K. , Dai, Q. , and Wei, C. 2016. “Physicochemical Properties of Indica‐japonica Hybrid Rice Starch From Chinese Varieties.” Food Hydrocolloids 63: 356–363. 10.1016/j.foodhyd.2016.09.013.

[jfds71534-bib-0053] Zuo, Y. , F. Zou , M. Yang , et al. 2024. “Effects of Plasma‐activated Water Combined With Ultrasonic Treatment of Corn Starch on Structural, Thermal, Physicochemical, Functional, and Pasting Properties.” Ultrasonics Sonochemistry 108: 106963. 10.1016/j.ultsonch.2024.106963. PMC11259921 38936293

